# Supplementary material for: Re-evaluation of the Bahariya Formation carcharodontosaurid (Dinosauria: Theropoda) and its implications for allosauroid phylogeny
Source: PLoS One. 2025 Jan 14;20(1):e0311096. doi: 10.1371/journal.pone.0311096 (PMC11731741; doi:10.1371/journal.pone.0311096)

Split set
Reduced strict consensus
equal weights

Strict consensus of 100000 trees

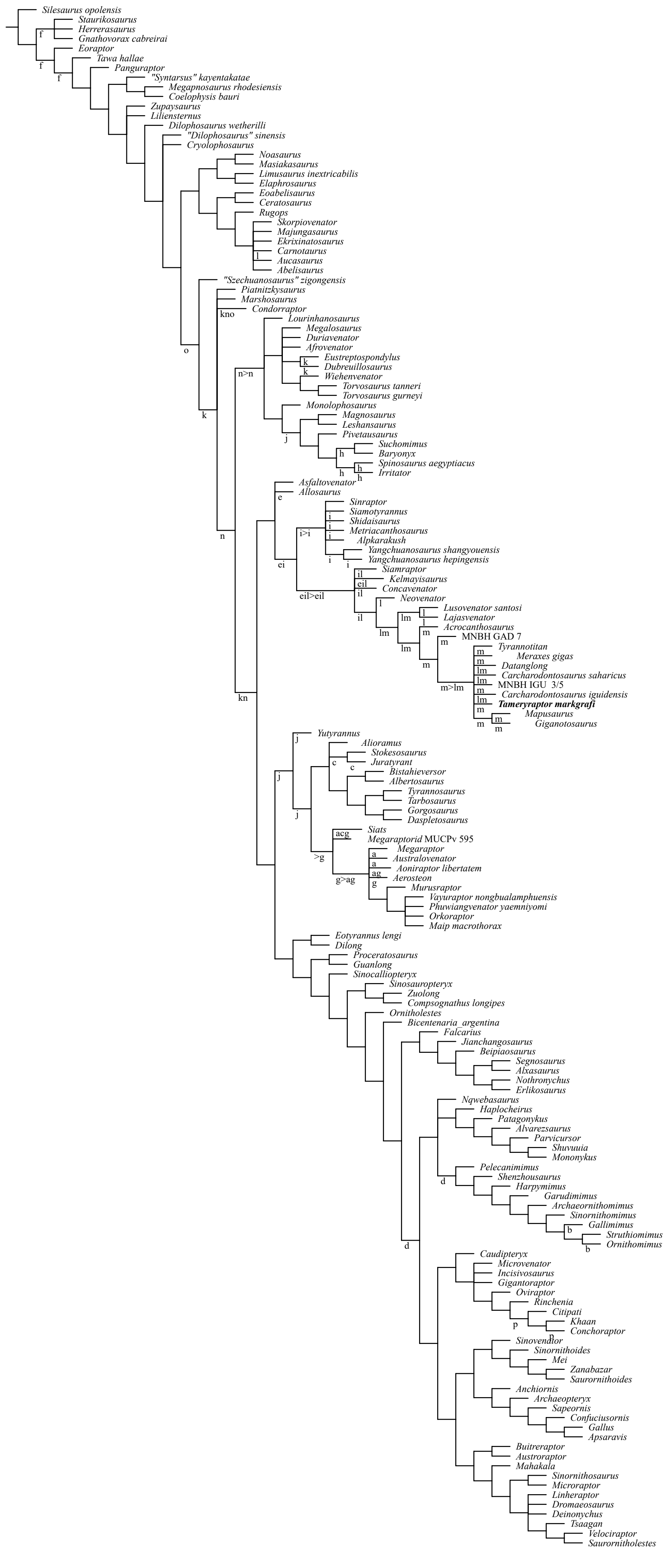

Legend:
a: MCNA PV 3139
b: *Anserimimus*
c: *Chilantaisaurus*
d: *Coelurus*
e: *Eocarcharia*
f: *Eodromaeus*
g: *Fukuiraptor*
h: *Ichthyovenator*
i: *Poekilopleuron*
j: *Shaochilong*
k: *Streptospondylus*
l: *Taurovenator*
m: *Veterupristisaurus*
n: *Xuanhanosaurus*
o: *Yunyangosaurus*
p: node 220 of consensus:

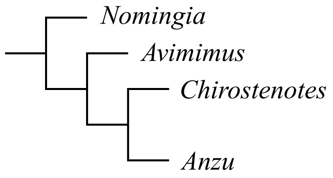

Split runs
Reduced strict consensus
$K = 15$

Legend:
a: *Aerosteon*
b: *Aoniraptor*
c: *Carcharodontosaurus iguidensis*
d: *Datanglong*
e: *Kelmayisaurus*
f: *Taurovenator*
g: *Veterupristisaurus*

Strict consensus of 58179 trees

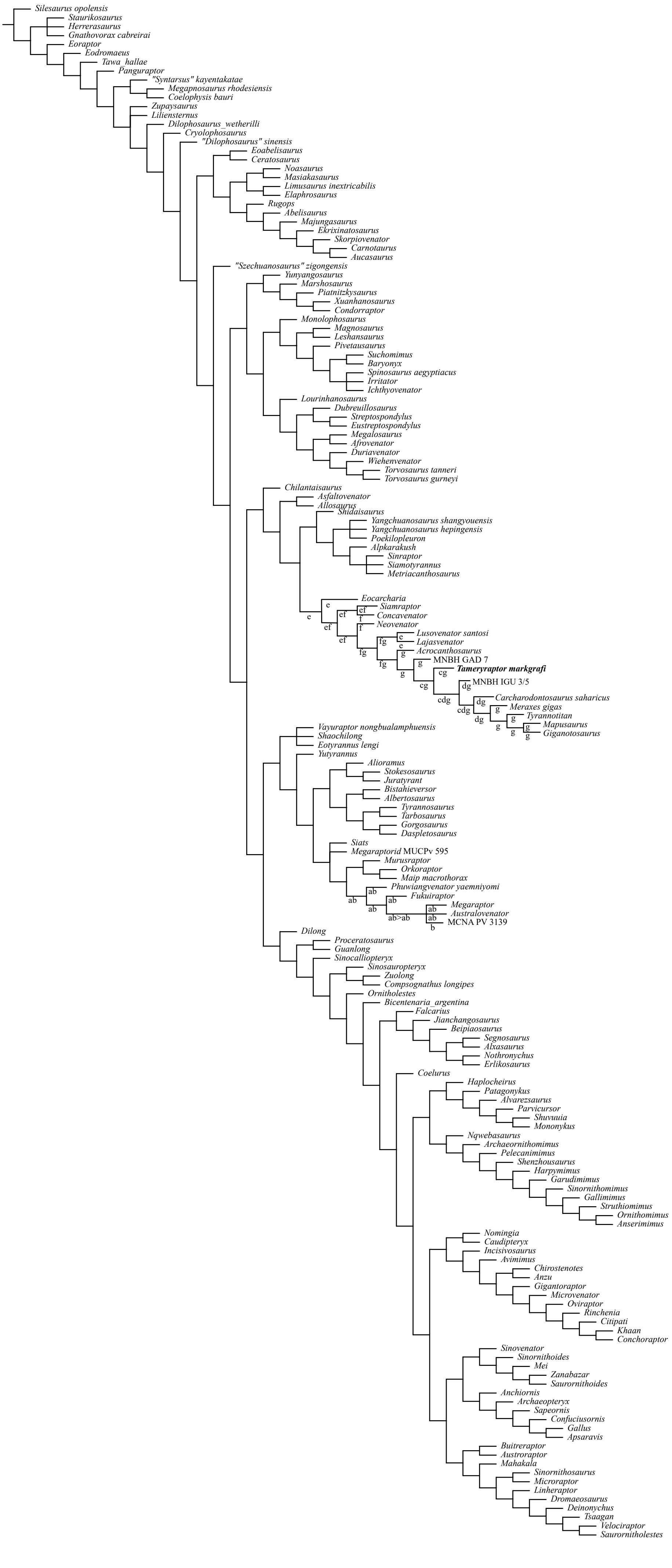

Split set
Reduced strict consensus
K = 12

Legend*:*
a: *Aerosteon*
b: *Aoniraptor*
c: *Carcharodontosaurus iguidensis*
d: *Kelmayisaurus*
e: *Taurovenator*
f: *Veterupristisaurus*

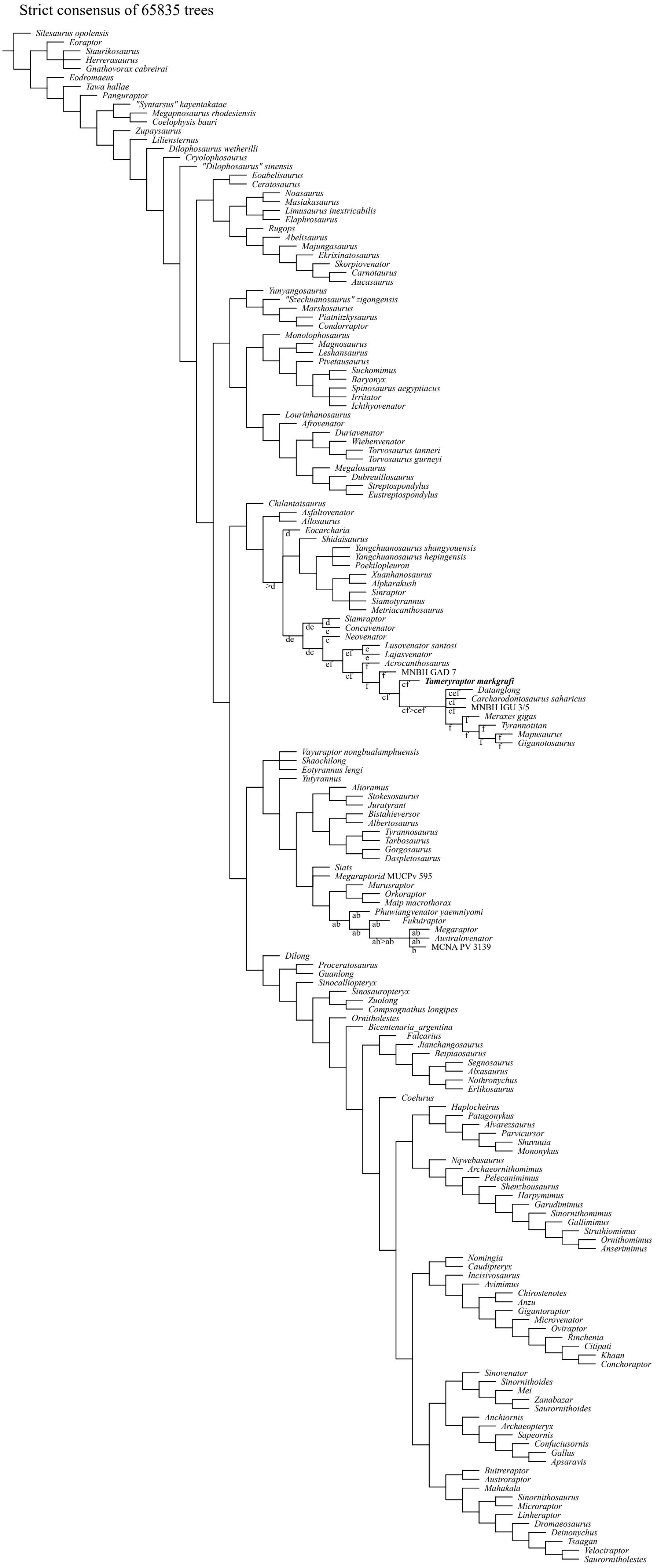

Split runs
Reduced strict consensus
K = 9

Legend:
a: *Aerosteon*
b: *Aoniraptor*
c: *Carcharodontosaurus iguidensis*
d: *Datanglong*
e: *Eocarcharia*
f: *Kelmayisaurus*
g: *Poekilopleuron*
h: *Streptospondylus*
i: *Veterupristisaurus*

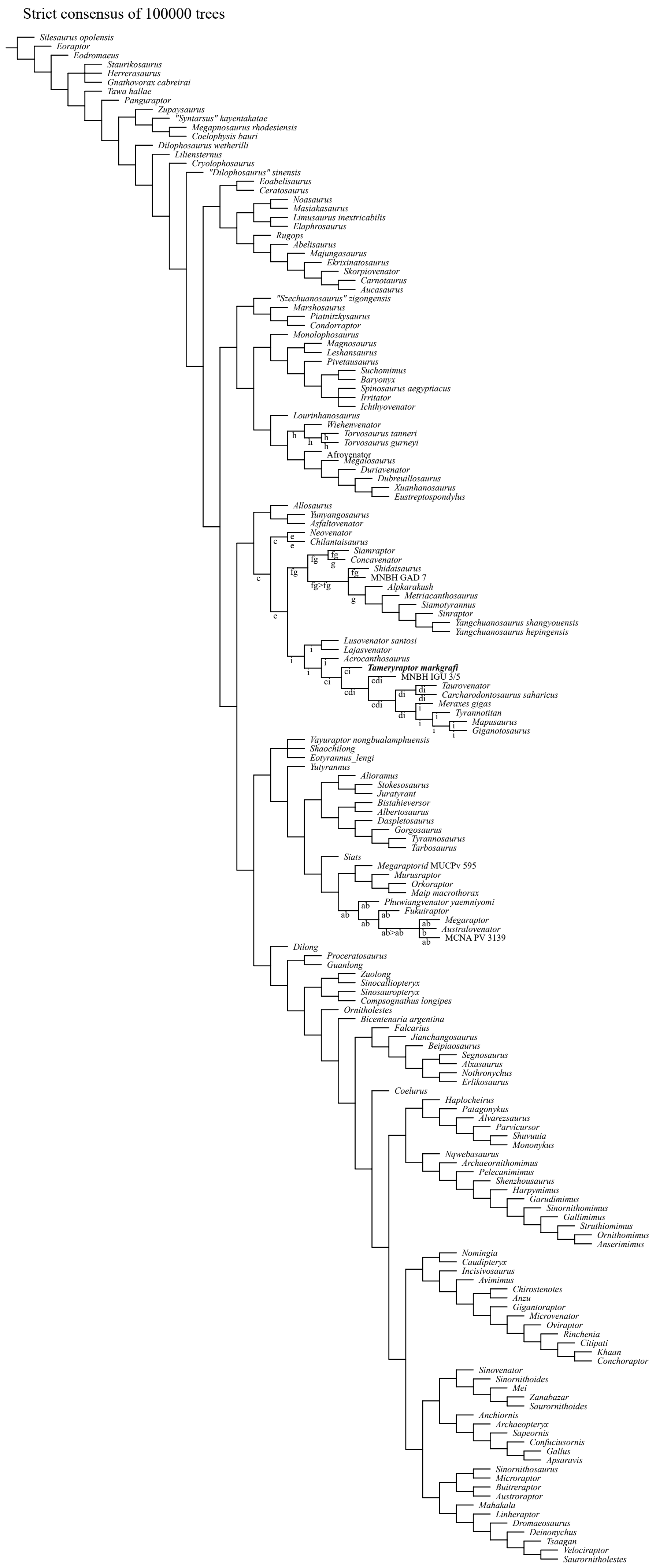

Merged set
Reduced strict consensus
equal weight

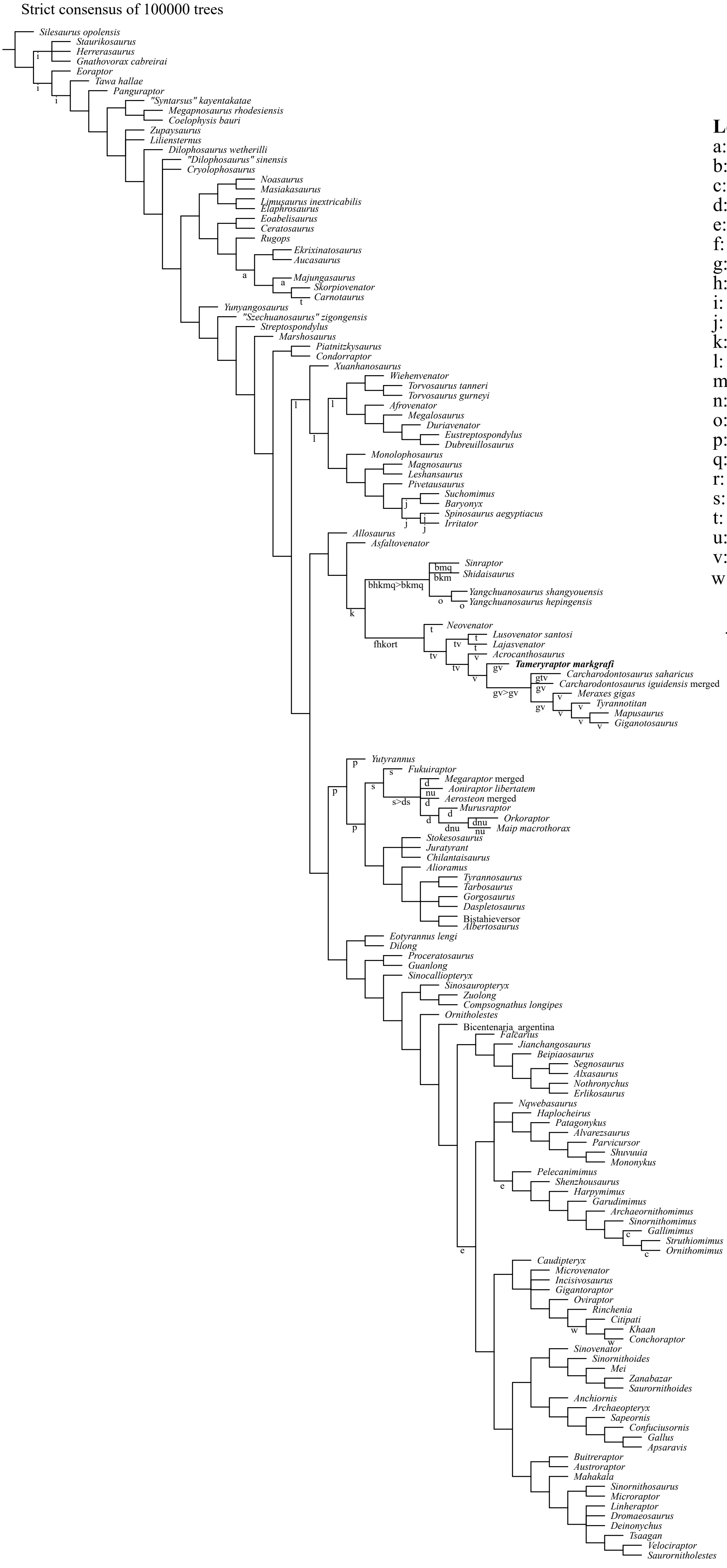

Legend:
a: *Abelisaurus*
b: *Alpkarakush*
c: *Anserimimus*
d: *Australovenator*
e: *Coelurus*
f: *Concavenator*
g: *Datanglong*
h: *Eocarcharia* merged
i: *Eodromaeus*
j: *Ichthyovenator*
k: *Kelmayisaurus*
l: *Lourinhanosaurus*
m: *Metriacanthosaurus*
n: *Phuwiangvenator yaemniyomi*
o: *Poekilopleuron*
p: *Shaochilong*
q: *Siamotyrannus*
r: *Siamraptor*
s: *Siats*
t: *Taurovenator*
u: *Vayuraptor nongbualamphuensis*
v: *Veterupristisaurus milneri*
w: node 221 of consensus

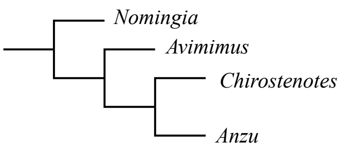

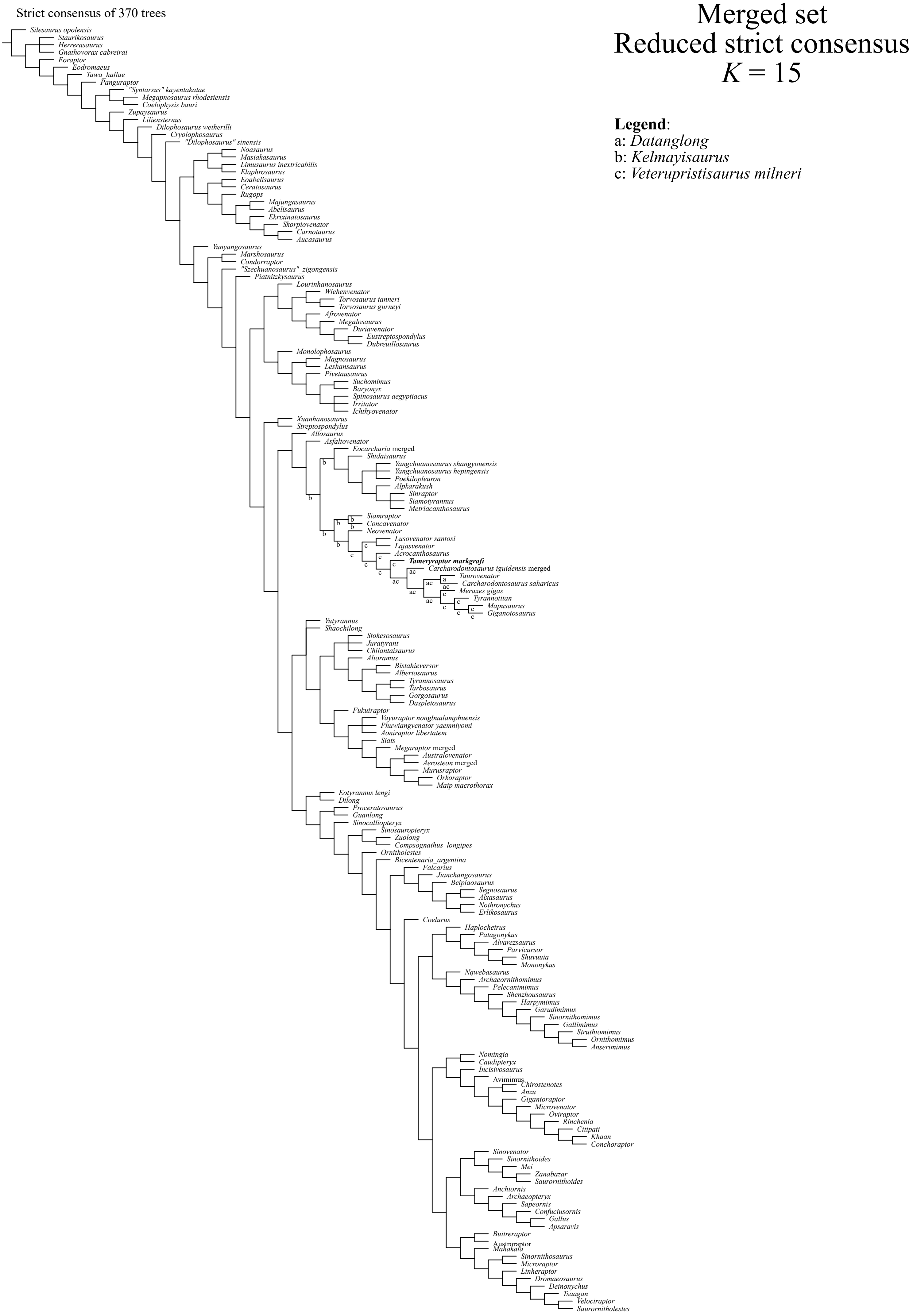
Strict consensus of 370 trees
Merged set
Reduced strict consensus
K = 15
Legend:
a: Datanglong
b: Kelmayisaurus
c: Veterupristisaurus milneri
Silesaurus opolensis
Staurikosaurus
Herrerasaurus
Gnathovorax cabreirai
Eoraptor
Eodromaeus
Tawa_hallae
Panguraptor
"Syntarsus" kayentakatae
Megapnosaurus rhodesiensis
Coelophysis bauri
Zupaysaurus
Liliensternus
Dilophosaurus wetherilli
Cryolophosaurus
"Dilophosaurus" sinensis
Noasaurus
Masiakasaurus
Limusaurus inextricabilis
Elaphrosaurus
Eoabelisaurus
Ceratosaurus
Rugops
Majungasaurus
Abelisaurus
Ekrixinatosaurus
Skorpiovenator
Carnotaurus
Aucasaurus
Yunyangosaurus
Marshosaurus
Condorraptor
"Szechuanosaurus"_zigongensis
Piatnitzkysaurus
Lourinhanosaurus
Wiehenvenator
Torvosaurus tanneri
Torvosaurus gurneyi
Afrovenator
Megalosaurus
Duriavenator
Eustreptospondylus
Dubreuillosaurus
Monolophosaurus
Magnosaurus
Leshansaurus
Pivetausaurus
Suchomimus
Baryonyx
Spinosaurus aegyptiacus
Irritator
Ichthyovenator
Xuanhanosaurus
Streptospondylus
Allosaurus
Asfaltovenator
Eocarcharia merged
Shidaisaurus
Yangchuanosaurus shangyouensis
Yangchuanosaurus hepingensis
Poekilopleuron
Alpkarakush
Sinraptor
Siamotyrannus
Metriacanthosaurus
Siamraptor
Concavenator
Neovenator
Lusovenator santosi
Lajasvenator
Acrocanthosaurus
Tameryraptor markgrafi
Carcharodontosaurus iguidensis merged
Taurovenator
Carcharodontosaurus saharicus
Meraxes gigas
Tyrannotitan
Mapusaurus
Giganotosaurus
Yutyrannus
Shaochilong
Stokesosaurus
Juratyrant
Chilantaisaurus
Alioramus
Bistahieversor
Albertosaurus
Tyrannosaurus
Tarbosaurus
Gorgosaurus
Daspletosaurus
Fukuiraptor
Vayuraptor nongbualamphuensis
Phuwiangvenator yaemniyomi
Aoniraptor libertatem
Siats
Megaraptor merged
Australovenator
Aerosteon merged
Murusraptor
Orkoraptor
Maip macrothorax
Eotyrannus lengi
Dilong
Proceratosaurus
Guanlong
Sinocalliopteryx
Sinosauropteryx
Zuolong
Compsognathus_longipes
Ornitholestes
Bicentenaria_argentina
Falcarius
Jianchangosaurus
Beipiaosaurus
Segnosaurus
Alxasaurus
Nothronychus
Erlikosaurus
Coelurus
Haplocheirus
Patagonykus
Alvarezsaurus
Parvicursor
Shuvuuia
Mononykus
Nqwebasaurus
Archaeornithomimus
Pelecanimimus
Shenzhousaurus
Harpymimus
Garudimimus
Sinornithomimus
Gallimimus
Struthiomimus
Ornithomimus
Anserimimus
Nomingia
Caudipteryx
Incisivosaurus
Avimimus
Chirostenotes
Anzu
Gigantoraptor
Microvenator
Oviraptor
Rinchenia
Citipati
Khaan
Conchoraptor
Sinovenator
Sinornithoides
Mei
Zanabazar
Saurornithoides
Anchiornis
Archaeopteryx
Sapeornis
Confuciusornis
Gallus
Apsaravis
Buitreraptor
Austroraptor
Mahakala
Sinornithosaurus
Microraptor
Linheraptor
Dromaeosaurus
Deinonychus
Tsaagan
Velociraptor
Saurornitholestes

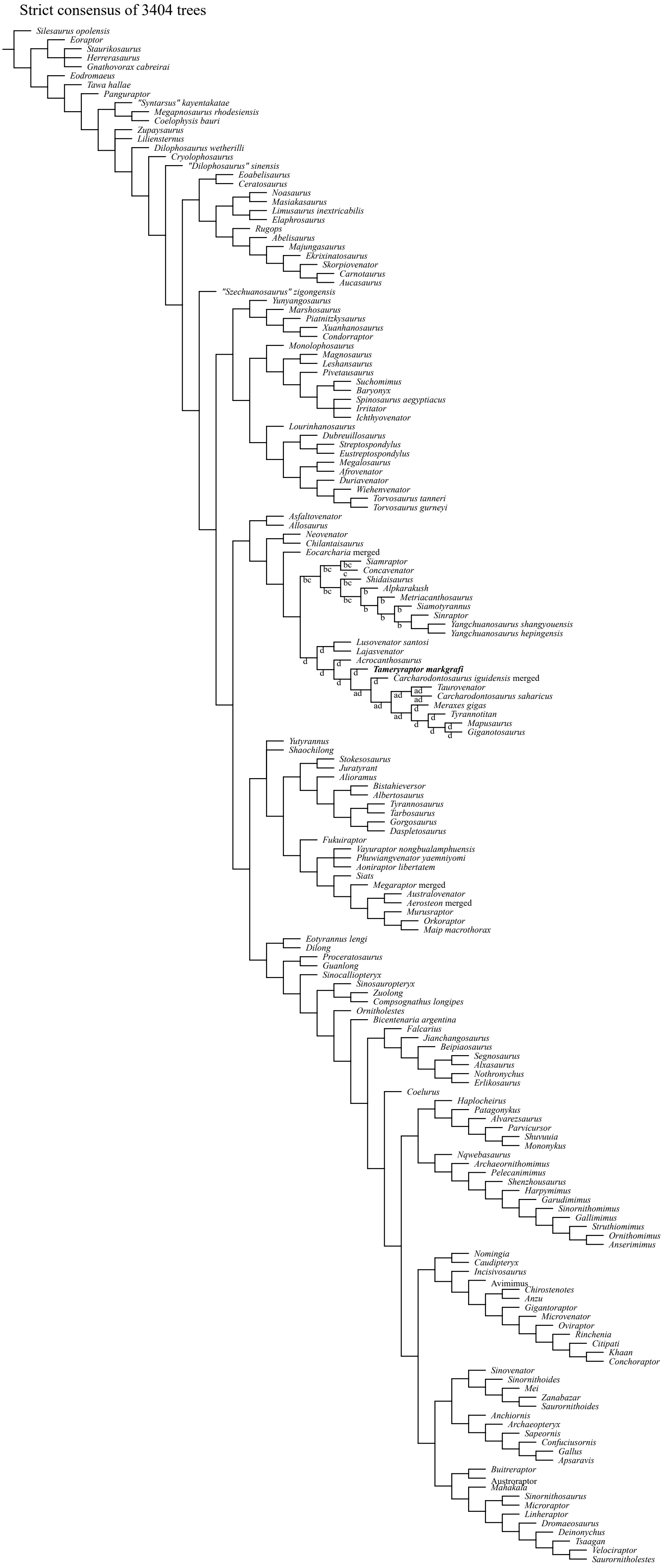

Merged set
Reduced strict consensus
K = 12

Legend:
a: *Datanglong*
b: *Kelmayisaurus*
c: *Poekilopleuron*
d: *Veterupristisaurus milneri*

Merged set
Reduced strict consensus
$K = 9$

Legend:
a: *Datanglong*
b: *Kelmayisaurus*
c: *Poekilopleuron*
d: *Streptospondylus*
e: *Veterupristisaurus milneri*

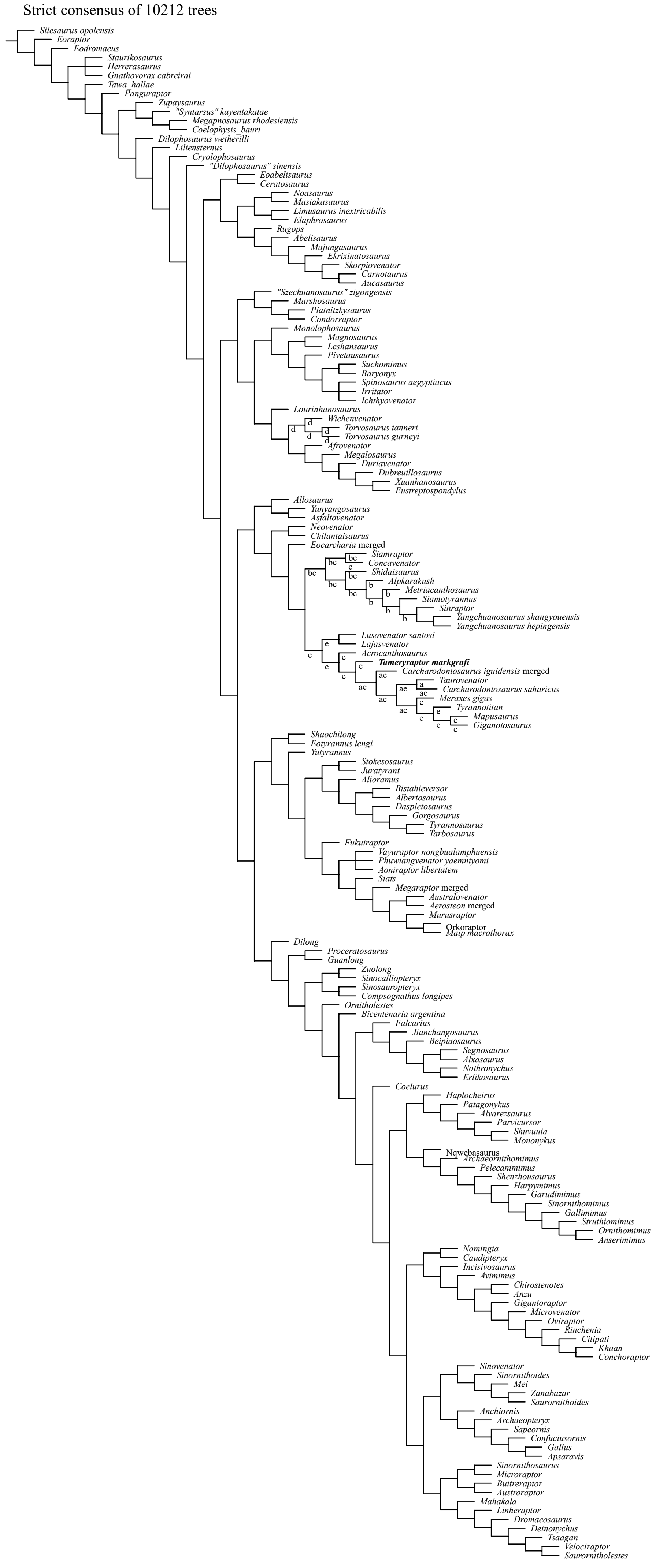

Supplement: S1 File — (PDF) [file pone.0311096.s002.pdf]
