# Supplementary material for: Re-evaluation of the Bahariya Formation carcharodontosaurid (Dinosauria: Theropoda) and its implications for allosauroid phylogeny
Source: PLoS One. 2025 Jan 14;20(1):e0311096. doi: 10.1371/journal.pone.0311096 (PMC11731741; doi:10.1371/journal.pone.0311096)
Supplement: S1 Table — * minimum measurement due to incompleteness of element;’ measured from photograph or drawings; Data taken from [1]. (DOCX) [file pone.0311096.s003.docx]

| **Element** | **Dimension measured** | **Measurement** |
| --- | --- | --- |
| **right Nasal** | Anteroposterior length | 540* |
|  | Lateromedial maximum width | ~80 |
|  | Lateromedial minimal width, at ½ anteroposterior length | 65 |
| **left Nasal** | Anteroposterior length | 450* |
|  | Lateromedial maximum width | ~80 |
|  | Lateromedial minimal width, at ½ anteroposterior length | 65 |
| **left Maxilla anterior fragment** | Anteroposterior length | ~470* |
|  | Dorsoventral height anteriorly | ~190* |
|  | Dorsoventral height posteriorly | ~70 |
| **left Maxilla posterior fragment** | Anteroposterior length | ~210* |
|  | Dorsoventral height anteriorly | 65 |
|  | Dorsoventral height posteriorly | ~25 |
| **10 anterior most preserved alveoli** | Anteroposterior length | ~40 |
|  | Lateromedial width | ~20 |
| **Most posterior alveolus** | Anteroposterior length | 25 |
|  | Lateromedial width | ~15 |
| **Maxillary tooth** | Dorsoventral height | 68 |
|  | Basal length | 35 |
|  | Number of denticles per cm | 18 - 20 |
| **Axis** | Centrum height anterior articular surface | 80 |
|  | Centrum width anterior articular surface | 110 |
|  | Neural canal height anterior | 25 |
|  | Neural canal width anterior | 28 |
| **4^th^ cervical vertebra “b”** | Centrum total length | 100 |
|  | Centrum length rim to rim | ~82**’** |
|  | Anterior centrum height | 68 |
|  | Anterior centrum width | 88 |
| **anterior caudal vertebra “d”** | Centrum total length | 145 |
|  | Centrum height anterior articular surface | 122 |
|  | Centrum width anterior articular surface | 122 |
| **Chevron “A”** | Transverse width dorsally | 75 |
|  | Shaft fragment proximodistal length | 130* |
| **Chevron “B”** | Transverse width dorsally | 65 |
|  | Proximodistal length | 150* |
| **Pubis** | Shaft length | >800* |
|  | Shaft diameter anteroposteriorly at ½ length | 75 |
| **Femur** | Proximodistal length | 1260 |
|  | Greatest lateromedial width proximal end | ~280 |
|  | Distance from proximal end to fourth trochanter | ~500 |
|  | Greatest lateromedial width distal end | ~260 |
| **Fibula** | Proximodistal length | 880 |
|  | Greatest anteroposterior width proximal end | 180 |
|  | Greatest lateromedial width proximal end | 75 |
|  | Shaft diameter at ½ height anteroposterior | 55 |
|  | Shaft diameter at ½ height lateromedial | 33 |
|  | Greatest anteroposterior width distal end | 58 |
|  | Greatest lateromedial width distal end | 109 |
