# Supplementary material for: Re-evaluation of the Bahariya Formation carcharodontosaurid (Dinosauria: Theropoda) and its implications for allosauroid phylogeny
Source: PLoS One. 2025 Jan 14;20(1):e0311096. doi: 10.1371/journal.pone.0311096 (PMC11731741; doi:10.1371/journal.pone.0311096)
Supplement: S2 Table — Unambiguous characters are listed with support under accelerated (ACCTRAN) and delayed (DELTRAN) transformations; autapomorphic values are not listed. (DOCX) [file pone.0311096.s004.docx]

| Node | unambiguous (split) | Acctran | Deltran |
| --- | --- | --- | --- |
| Node (1): Allosauroidea | 584: (2) → (1), 681: (0) → (1) | 66: (1) → (0), 72: (0) → (1), 89: (0) → (1), 92: (0) → (1), 114: (1) → (0), 126: (1) → (2),  238: (0) → (1), 251: (0) → (1), 269: (0) → (1), 346: (0) → (1), 363: (2) → (1), 421: (1) → (0), 458: (0) → (1), 511 (1) → (2), 512 (0) → (1), 515 (0) → (1), 624: (0) → (1), 706: (0) → (1), 710: (0) → (1), 745: (0) → (1) |  |
| Node (2) | 577: (1) → (0), 816: (0) → (1) | 491: (3) → (4), 660: (0) → (1) | 83: (1) → (0), 116: (0) → (1), 126: (1) → (2), 129: (0) → (1), 145: (0) → (1), 203: (1) → (2), 209: (0) → (1), 251: (0) → (1), 512: (0) → (1), 547: (0) → (1), 560: (0) → (1), 610: (0) → (1), 612: (0) → (1), 637: (0) → (1), 668: (0) → (1), 746: (1) → (0) |
| Node (3):  Allosauridae | 32: (1) → (0), 82: (01) → (1), 120: (0) → (1) 186: (0) → (1), 334: (0) → (1), 569: (0) → (1), 570: (0) → (1) | 70: (0) → (1), 151: (0) → (1), 169: (1) → (2), 263: (1) → (2), 289: (1) → (0), 318: (0) → (1), 449: (0) → (1), 516: (1) → (0), 680: (0) → (1), 708: (1) → (2), 744: (1) → (2) | 13: (0) → (1), 66: (1) → (0), 72: (0) → (1), 245: (0) → (1), 346: (0) → (1), 624: (0) → (1) |
| Node (4):  Carcharodontosauriformes | 172: (0) → (1), 194: (0) → (1), 196: (1) → (2), 219: (0) → (1), 264: (0) → (1), 376: (3) → (2), 377: (3) → (2), 379: (0) → (1), 406: (0) → (1), 508: (0) → (1), 576: (0) → (1), 605: (0) → (1), 609: (0) → (1) | 90: (1) → (0), 135: (0) → (1), 159: (0) → (1), 165: (0) → (1), 171: (0) → (1), 325: (0) → (1), 474: (1) → (2), 525: (0) → (1), 579: (0) → (1,2), 598: (0) → (1), 639: (2) → (1), 663: (0) → (1) | 92: (0) → (1), 199: (0) → (1), 238 (0) → (1), 290: (1) → (0); 304: (1) → (0), 329: (0) → (1), 706: (0) → (1), |
| Node (6):  Metriacanthosauridae | 252: (1) → (0), 300: (1) → (0), 402: (1) → (0), 407: (1) → (0), 655: (0) → (1), 662: (2) → (0), 669: (0) → (1), 681: (1) → (0), 695: (0) → (1), 696: (1) → (0), 716: (0) → (1) | 20: (1) → (2), 37: (0) → (2), 38: (1) → (2), 43: (1) → (0), 81: (0) → (1), 96: (0) → (1), 113: (0) → (1), 134: (1) → (2), 137: (0) → (1), 146: (0) → (1), 173: (0) → (2), 174: (0) → (1), 191: (0) → (1), 237: (0) → (3), 273: (1) → (0), 284: (1) → (0), 291: (0)→ (1), 294: (0) → (1), 302: (0) → (1), 316: (1) → (2), 370: (1) → (0), 378: (0) → (1), 434: (0) → (1), 460: (0) → (1), 626: (2) → (1), 648: (0) → (1), 650: (1) → (0), 651: (0) → (1), 664: (1) → (0), 670: (0) → (1), 674: (0) → (1), 712: (1) → (0), 737: (0) → (1), 797: (0) → (1) | 663: (0) → (1), 693: (0) → (1) |
| Node (7):  Carcharodontosauria | 52: (2) → (1), 60: (0) → (1), 152: (0) → (1), 157: (0) → (1), 445: (0) → (1), 562: (0) → (1), 581: (0) → (2), 738: (1) → (2), 775: (0) → (1), 785: (1) → (0) | 63: (0) → (1), 64: (1) → (2), 73 (0) → (1), 88: (0) → (1), 144: (1) → (2), 155: (0) → (1), 164: (0) → (1), 184: (0) → (1), 204: (0) → (1), 217: (0) → (1),  221: (0) → (1), 229: (0) → (1), 247: (1) → (2), 260: (0) → (1), 263: (1) → (0), 270: (0) → (1), 274: (1) → (0), 282: (0) → (1), 313: (0) → (1), 329: (0) → (1), 332: (0) → (1), 414: (1) → (0), 624: (1) → (0), 657: (0) → (1), 660: (0) → (1),  687: (0) → (1), 758: (0) → (1), 768: (2) → (1), 801: (0) → (1), 808: (1) → (0) | 166: (1) → (0), 415: (0) → (1), 421: (1) → (0), 525: (0) → (1), 558: (0) → (1), 579: (0) → (1), 657: (0) → (1), 665: (0) → (1), 692: (1) → (0) |
| Node (8) | 55: (1) → (0), 396: (0) → (1) | 161: (0) → (2), 357: (0) → (2), 513: (0) → (2), 522: (1) → (2), 583: (1) → (0), 586: (0) → (1), 595: (0) → (1), 597: (1) → (0), 602: (2) → (1), 615: (1) → (0), 634: (1) → (2), 680: (0) → (1), 821: (2) → (1) | 511: (1,2) → (1) |
| Node (9) | 8: (0) → (1), 74: (0) → (1), 417: (1) → (2), 422: (0) → (1), 452: (1) → (2), 469: (0) → (1), 496: (2) → (1), 744: (1) → (2) | 56: (1) → (0), 76 (0) → (1), 82 (0) → (1), 173: (0) → (1), 222: (2) → (3), 466: (0) → (1), 474: (2) → (1), 520: (0) → (1), 570: (0) → (1), 587: (1) → (2), 635: (0) → (1), 643: (1) → (0), 741: (0) → (1), 768: (1) → (2) | 313: (0) → (1), 320: (1) → (0), 515: (0) → (1), 710: (0) → (1) |
| Node (10):  Carcharodontosauridae | 308: (0) → (1), 460: (0) → (1), 483: (0) → (1), 510: (0) → (1), 511: (1) → (0), 688: (0) → (1) | 33: (0) → (1), 56: (0) → (1), 80 (0) → (1), 386: (0) → (1), 509: (0) → (1), 657: (1) → (0), 712: (1) → (2), 747: (0) → (1), 784: (1) → (0) | 159: (0) → (1), 160: (0) → (1), 520: (0) → (1), 691: (1) → (0), 715 (0) → (1), 729: (0) → (1) |
| Node (11) | 433: (0) → (1), 525: (1) → (0), 675: (0) → (1) | 61: (0) → (1), 352: (01) → (0), 447: (1) → (0),  448: (0) → (1), 493: (0) → (1), 655: (0) → (1), 668: (1) → (0), 673: (0) → (1), 693: (0) → (1), 748: (0) → (1), 831: (0) → (1) | 509: (0) → (1) |
| Node (12) | 65: (1) → (2), 113: (0) → (1), 418: (1) → (0), 450: (0) → (1), 531: (1) → (0), 708: (1) → (3) | 118: (0) → (1), 374: (0) → (1), 386: (1) → (0), 509: (1) → (0), 516: (1) → (0), 657: (0) → (1), 712: (2) → (1), 747: (1) → (0) | 80 (0) → (1), 82 (0) → (1),  112: (0) → (1), 135: (0) → (1), 144: (1) → (2), 155: (0) → (1), 164: (0) → (1), 171: (0) → (1), 173: (0) → (1), 184: (0) → (1), 199: (0) → (1), 204: (0) → (1), 217: (0) → (1), 222: (2) → (3), 230: (1) → (0), 247: (1) → (2), 260: (0) → (1), 269: (0) → (1), 270: (0) → (1), 466: (0) → (1), 474: (1) → (2), 570: (0) → (1), 587: (1) → (2), 639: (2) → (1) |
| Node (13) | 37: (0) → (2), 378: (0) → (1) | 43: (1) → (0), 72 (0) → (1,2), 84 (0) → (2), 97: (0) → (1), 105: (0) → (1), 119: (0) → (1), 125: (1) → (0), 147: (0) → (1), 148: (2) → (0), 169: (0) → (1), 190: (0) → (1), 201: (1) → (0), 202: (0) → (1), 210: (1) → (0), 236: (2) → (3), 254: (1) → (0), 272: (1) → (0),  277: (1) → (0), 285: (2) → (1), 293: (0) → (1), 296: (0) → (1), 309: (0) → (1), 310: (0) → (1), 311: (0) → (1), 327: (1) → (0), 328: (0) → (1), 353: (1) → (2),  386: (0) → (1), 421: (0) → (1), 496: (2) → (1), 507: (0) → (1), 509: (0) → (1), 554: (1) → (0), 562: (1) → (0), 592: (0) → (1), 639: (1) → (0), 712: (1) → (2), 741: (1) → (0), 747: (0) → (1), 768: (2) → (1), 771: (0) → (1), 782: (0) → (1), 785: (0) → (1), 805: (0) → (1), 826: (1) → (0) |  |
| Node (14) | 52: (1) → (0), 55: (1) → (0), 363: (2) → (1), 396: (0) → (1) | 27: (2) → (0), 72 (2) → (1) | 76 (0) → (1), 84 (0) → (2), 147: (0) → (1), 148: (2) → (0), 293: (0) → (1), 507: (0) → (1), 516: (1) → (0), 758: (0) → (1), 782: (0) → (1), 784: (1) → (0) |
| *Tameryraptor* | 82 : (1) → (0), 85: (0) → (1), 87: (1) → (0), 92: (1) → (0), 252: (1) → (0), 258: (0) → (1), 377: (2) → (1), 378: (1) → (2), 379: (1) → (2), 446: (1) → (0), 493: (0) → (2), 501: (1) → (2), 504: (0) → (1), 700: (1) → (0), 715: (1) → (0), 782: (1) → (2) |  | 496: (2) → (1), 712: (1) → (2), 785: (0) → (1) |
| Node (15):  Carcharodontosaurinae | 95: (0) → (1), 301: (0) → (1), 302: (0) → (1), 370: (1) → (0), 501: (1) → (0) | 154: (0) → (1), 256: (0) → (1), 401: (0) → (1), 426: (0) → (1), 726: (1) → (0) | 72 (2) → (1), 165: (0) → (1), 169: (0) → (1), 263: (1) → (0), 272: (1) → (0), 285: (2) → (1), 296: (0) → (1), 309: (0) → (1), 310: (0) → (1), 311: (0) → (1), 386: (0) → (1), 657: (0) → (1) |
| Node (18):  Giganotosaurini | 39: (0) → (1), 265: (0) → (1), 269: (1) → (0), 456: (1) → (0), 680: (0) → (1) | 53: (0) → (1), 123 (0) → (1), 372: (0) → (1), 378: (1) → (0) | 43: (1) → (0), 97: (0) → (1), 154: (0) → (1), 202: (0) → (1), 277: (1) → (0), 290: (1) → (0), 747: (0) → (1) |
| Node (19) | 108: (1) → (0), 493: (0) → (1), 544: (1) → (0), 708: (3) → (1) | 33: (1) → (0), 37: (2) → (0), 208: (1) → (0), 323: (0) → (1), 421: (1) → (0), 547: (1) → (0), 576: (0) → (1), 650: (1) → (2), 662: (2) → (0), 680: (1) → (0), 712: (2) → (1), 726: (0) → (1) | 426: (0) → (1) |
| Node (20) | 320: (0) → (1), 741: (0) → (1), 744: (2) → (1) |  | 37: (0) → (2), 208: (1) → (0), 328: (0) → (1), 374: (0) → (1), 501: (1) → (0), 657: (0) → (1), 726: (1) → (0) |
| Node (21):  Coelurosauria | 39: (0) → (1), 85: (0) → (1), 157: (0) → (1), 181: (0) → (2), 187: (1) → (0), 198: (1) → (0), 205: (0) → (1), 222: (2) → (1), 232: (0) → (1), 241: (2) → (1), 274: (1) → (0), 279: (1) → (0), 294: (0) → (1), 295: (2) → (1), 340: (0) → (1), 357: (0) → (2), 374: (0) → (1), 513: (0) → (3), 532: (0) → (1), 536: (1) → (0), 607: (1) → (0), 621: (0) → (1), 661: (0) → (1), 673: (0) → (12), 670: (0) → (1), 726: (1) → (0), 776: (0) → (1), 795: (1) → (0) | 121: (0) → (1), 203: (0) → (1), 213: (0) → (1), 286: (1) → (0), 305: (1) → (0), 367: (0) → (1), 479: (0) → (1), 504: (0) → (1), 524: (0) → (1), 583: (1) → (0), 585: (0) → (1), 619: (1) → (0), 641: (0) → (1), 678: (0) → (1) | 558: (0) → (1), 746: (0) → (1) |
| Node (22):  Tyrannosauroidea | 52: (2) → (1), 74: (0) → (1), 137: (0) → (1),  183: (0) → (1), 208: (1) → (0), 328: (0) → (1), 483: (0) → (1), 511: (1) → (0), 525: (0) → (1), 775: (0) → (1), 793: (0) → (1), 797: (0) → (1) | 38: (1) → (2),  67: (1) → (0), 79: (0) → (1), 105: (0) → (1), 124: (1) → (3), 182: (0) → (1), 191: (0) → (2), 194: (0) → (2), 227: (0) → (1), 237: (0) → (1), 241: (1) → (0), 414 (1) → (2), 445: (0) → (1), 462: (0) → (1), 541: (0) → (1), 672: (0) → (1), 688: (0) → (1) | 124: (1) → (2) |
| Node (23) | 43: (1) → (0), 60: (0) → (1), 771: (0) → (1) | 27: (2) → (0), 54: (0) → (1),  65: (1) → (2), 72: (0) → (1), 150: (0) → (1), 152: (0) → (1), 155: (0) → (1), 263: (1) → (0), 285: (2) → (3),  290: (1) → (2), 321: (1) → (0), 376: (2) → (1), 456: (1) → (2), 505: (0) → (1), 613: (1) → (0), 632: (0) → (1) |  |
| Node (24) | 84: (0) → (2), 91: (1) → (0), 620: (1) → (2) | 4: (3) → (4), 8: (0) → (1), 80: (0) → (1), 88: (0) → (1), 149: (0) → (1), 151: (0) → (1), 203: (0) → (1), 225: (0) → (1), 252: (1) → (0), 263: (1) → (2), 264: (0) → (1),  286: (0) → (1),  290: (1) → (0),  291: (0) → (1), 302: (0) → (1), 323: (0) → (1), 409: (1) → (0), 543: (0) → (1), 773: (0) → (1) | 191: (0) → (2), 194: (0) → (2), 209: (0) → (1), 693 (1) → (0), |
| Node (25) | 48: (0) → (1), 75: (0) → (1), 84: (1) → (0), 86: (1) → (0), 451: (1) → (2) | 4: (4) → (3), 8: (1) → (0), 104: (0) → (1), 129: (0) → (1), 161: (0) → (2), 190: (0) → (2), 224: (1) → (0), 363: (1) → (2), 445: (0) → (1) | 38: (1) → (2), 67: (1) → (0), 149: (0) → (1), 151: (0) → (1), 166: (0) → (1), 190: (0) → (1), 225: (0) → (1), 237: (0) → (1), 241: (1) → (0), 263: (1) → (2), 290: (1) → (0), 291: (0) → (1), 302: (0) → (1), 330: (1) → (0), 399 (1) → (2), 715: (1) → (0), 773: (0) → (1) |
| Node (26):  Megaraptora | 52: (1) → (2), 285: (2) → (0), 294: (1) → (0), 296: (0) → (1), 373: (0) → (1), 425: (1) → (2), 493: (0) → (2), 501: (1) → (2), 502: (1) → (2), 506: (0) → (1), 545: (0) → (1), 574: (0) → (1), 584:(2) → (1), 668: (0) → (1) | 11: (1) → (0), 64: (1) → (2), 74: (1) → (0), 81: (0) → (1), 120: (0) → (1), 141: (0) → (1), 158: (0) → (1), 164: (0) → (1), 168: (0) → (1), 170: (1) → (0), 176: (0) → (1), 179: (0) → (1), 180: (1) → (0), 182: (1) → (0), 208: (0) → (2), 239: (2) → (0), 254: (1) → (0), 299: (0) → (1), 321: (1) → (0), 346: (0) → (1), 353: (1) → (0), 355: (0) → (1), 414: (1) → (0), 447: (1) → (0), 586: (0) → (1), 614: (0) → (1), 630: (0) → (1),  631: (0) → (1), 637: (0) → (1), 640: (1) → (0), 660: (0) → (1), 662: (2) → (0), 673: (2) → (0), 808: (1) → (0) | 252: (1) → (0), 417 (2) → (1), 504: (0) → (1), 543: (0) → (1), 560 (0) → (1), 583: (1) → (0), 692: (1) → (0), 785: (1) → (0) |
