# Supplementary material for: Re-evaluation of the Bahariya Formation carcharodontosaurid (Dinosauria: Theropoda) and its implications for allosauroid phylogeny
Source: PLoS One. 2025 Jan 14;20(1):e0311096. doi: 10.1371/journal.pone.0311096 (PMC11731741; doi:10.1371/journal.pone.0311096)
Supplement: S3 Table — Unambiguous characters are listed with support under accelerated (ACCTRAN) and delayed (DELTRAN) transformations. Autapomorphic values are not listed. (DOCX) [file pone.0311096.s005.docx]

| Node | unambiguous | ACCTRAN | DELTRAN |
| --- | --- | --- | --- |
| Node (1):  Allosauroidea | 61: (1) → (0), 66: (1) → (0), 82: (0) → (1), 86: (0) → (1), 114: (1) → (0), 126: (1) → (2), 148: (0) → (2), 236: (4) → (2); 251: (0) → (1), 512: (0) → (1), 515: (0) → (1), 543: (0) → (1), 560: (0) → (19, 610: (0) → (1),     612: (0) → (1), 681: (0) → (1), 710: (0) → (1), 744: (1) → (2) | 89: (0) → (1); 230: (1) → (0), 238: (0) → (1), 269: (1) → (0), 313: (0) → (1), 346: (0) → (1), 363: (1) → (2), 421: (1) → (0), 423: (1) → (2), 458: (0) → (1), 479: (0) → (1), 577: (1) → (0), 706: (0) → (1) | 547: (0) → (1), 637: (1) → (2) |
| Node (2):  Allosauridae | 72: (0) → (1), 120: (0) → (1), 186: (0) → (1), 334: (0) → (1), 569: (0) → (1), 570: (0) → (1) | 70: (0) → (1); 151: (0) → (1), 169: (1) → (2), 289: (1) → (0), 318: (0) → (1), 320: (0) → (1), 449: (0) → (1), 479: (1) → (0), 511: (1) → (2), 516: (1) → (0), 660: (0) → (1), 680: (0) → (1), 693: (0) → (1), 708: (1) → (2) | 215: (0) → (1); 230: (1) → (0); 346: (0) → (1), 417: (2) → (1) |
| Node (3) | 52: (2) → (1), 64: (1) → (2), 406: (0) → (1), 445: (0) → (1), 665: (0) → (1), 738: (1) → (2), 775: (0) → (1) | 135: (0) → (1), 171: (0) → (1), 172: (0) → (1), 175: (1) → (2), 194: (0) → (1), 196: (1) → (2); 219: (0) → (1), 290: (1) → (0), 325: (0) → (1), 377: (3) → (2), 378: (0) → (1), 379: (0) → (1), 422: (0) → (1), 452: (1) → (2), 469: (0) → (1), 496: (1) → (2), 562: (0) → (1), 598: (0) → (1), 605: (0) → (1), 609: (0) → (1), 729: (1) → (0) | 415: (0) → (1), 421: (1) → (0), 423: (1) → (2), 458: (0) → (1), 525: (0) → (1), 549: (1) → (2) |
| Node (4) | 768: (2) → (1), 773: (0) → (1) | 4: (2) → (1), 9: (1) → (2), 11: (1) → (0); 20: (1) → (2), 21: (1) → (0); 38: (1) → (2); 63: (0) → (1), 74: (0) → (1), 88: (0) → (1); 350: (1) → (2); 352: (1) → (2), 414: (1) → (0), 456: (1) → (0), 462: (0) → (1), 472: (0) → (1), 473: (0) → (1), 484: (0) → (1), 501: (1) → (0), 523: (0) → (1), 571: (1) → (0), 575: (0) → (1), 640: (0) → (1), 657: (0) → (1), 687: (0) → (1), 720: (0) → (1), 758: (0) → (1), 765: (0) → (1), 783: (1) → (2), 808: (1) → (0) | 15: (1) → (0) |
| Node (5) | 37: (0) → (2), 73: (0) → (1) | 13: (1) → (0), 92: (0) → (1), 452: (2) → (1), 460: (0) → (1), 469: (1) → (0), 474: (1) → (2), 508: (0) → (1), 510: (0) → (1), 576: (0) → (1), 579: (0) → (1), 581: (0) → (2), 639: (2) → (1), 688: (0) → (1) | 32: (0) → (1), 171: (0) → (1), 175: (1) → (2), 581: (0) → (1) |
| Node (6):  Carcharodontosauriformes | 199: (0) → (1) | 96: (0) → (1), 152: (0) → (1), 157: (0) → (1) | 92: (0) → (1), 194: (0) → (1), 196: (1) → (2), 219: (0) → (1); 238: (0) → (1), 412: (0) → (1), 479: (0) → (1), 508: (0) → (1), 579: (0) → (1), 581: (1) → (2), 706: (0) → (1), 816: (0) → (1) |
| Node (7):  Metriacanthosauridae | 82: (1) → (0), 160: (1) → (0), 304: (1) → (0), 660: (0) → (1), 663: (0) → (1), 744: (2) → (1) | 64: (2) → (1); 66: (0) → (1), 109: (2) → (1), 146: (0) → (1), 174: (0) → (1), 191: (0) → (1); 237: (0) → (3), 252: (1) → (0), 273: (1) → (0); 284: (1) → (0), 291: (0) → (1), 294: (0) → (1), 300: (1) → (0), 302: (0) → (1), 316: (1) → (2), 320: (0) → (1), 407: (1) → (0), 448: (0) → (1), 511: (1) → (2), 515: (1) → (0), 583: (1) → (0), 586: (0) → (1), 595: (0) → (1), 597: (1) → (0), 602: (2) → (1), 615: (1) → (0), 621: (0) → (1), 634: (1) → (2), 674: (0) → (1), 695: (0) → (1), 696: (1) → (0), 710: (1) → (0), 745: (1) → (0), 770: (0) → (1), 797: (0) → (1), 829: (0) → (1) | 8: (1) → (0), 377: (3) → (2), 452: (2) → (1), 460: (1) → (0), 474: (1) → (2) |
| Node (8) | 55: (1) → (0), 396: (0) → (1) | 23: (1) → (0); 63: (0) → (1), 129: (1) → (2), 161: (0) → (2), 332: (0) → (1); 357: (0) → (2), 418: (1) → (0), 458: (1) → (0), 522: (1) → (2), 680: (0) → (1), 821: (2) → (1) | 715: (0) → (1) |
| Node (9) | 402: (1) → (0), 655: (0) → (1), 662: (2) → (0), 681: (1) → (0), 693: (0) → (1), 712: (1) → (0), 716: (0) → (1) | 20: (1) → (2); 38: (1) → (2); 43: (1) → (0); 52: (2) → (3); 60: (1) → (0); 72: (0) → (1), 81: (0) → (1), 134: (1) → (2), 137: (0) → (1), 173: (0) → (2), 175: (2) → (1), 300: (1) → (0), 329: (1) → (0), 370: (1) → (0), 434: (0) → (1), 445: (1) → (0), 513: (0) → (2), 515: (0) → (1), 562: (1) → (0), 626: (2) → (1), 648: (0) → (1), 651: (0) → (1), 668: (1) → (0), 690: (0) → (1), 737: (1) → (0), 775: (1) → (0) | 252: (1) → (0), 407: (1) → (0), 460: (0) → (1), 696: (1) → (0), 729: (1) → (0) |
| Node (10):  Carcharodontosauridae | 74: (0) → (1), 308: (0) → (1), 483: (0) → (1), 511: (1) → (0), 520: (0) → 1 | 76: (0) → (1), 80: (0) → (1); 144: (1) → (2), 164: (0) → (1); 173: (0) → (1), 184: (0) → (1), 204: (0) → (1), 217: (0) → (1), 221: (0) → (1), 222: (2) → (3); 229: (0) → (1); 260: (0) → (1), 263: (1) → (0); 270: (0) → (1), 274: (1) → (0); 282: (0) → (1), 386: (0) → (1), 466: (0) → (1), 469: (0) → (1),  509: (0) → (1), 570: (0) → (1), 587: (1) → (2),  635: (0) → (1), 643: (1) → (0), 712: (1) → (2), 729: (0) → (1), 747: (0) → (1), 784: (1) → (0), 801: (0) → (1) | 417: (1) → (2), 422: (0) → (1), 460: (0) → (1), 496: (1) → (2), 510: (0) → (1), 688: (0) → (1), 691: (1) → (0), 715: (0) → (1) |
| Node (11) | 433: (0) → (1), 525: (1) → (0), 675: (0) → (1) | 61: (0) → (1), 351: (1) → (0); 352: (1) → (0), 447: (1) → (0), 474: (2) → (1), 493: (0) → (1), 655: (0) → (1), 665: (1) → (0), 668: (1) → (0), 673: (0) → (1), 693: (0) → (1), 748: (0) → (1), 808: (1) → (0), 831: (0) → (1) | 509: (0) → (1) |
| Node (12) | 65: (1) → (2), 113: (0) → (1), 418: (1) → (0), 450: (0) → (1), 531: (1) → (0), 708: (1) → (3) | 118: (0) → (1), 184: (0) → (1), 374: (0) → (1), 414: (1) → (0), 462: (0) → (1), 509: (1) → (0), 516: (1) → (0), 657: (0) → (1), 680: (0) → (1), 712: (2) → (1), 747: (1) → (0) | 61: (0) → (1), 80: (0) → (1), 112: (0) → (1), 135: (0) → (1), 144: (1) → (2), 152: (0) → (1), 157: (0) → (1), 164: (0) → (1), 171: (0) → (1), 204: (0) → (1), 217: (0) → (1); 222: (2) → (3); 230: (1) → (0), 260: (0) → (1); 269: (0) → (1), 270: (0) → (1), 313: (0) → (1), 452: (1) → (2), 466: (0) → (1), 469: (0) → (1), 474: (1) → (2), 570: (0) → (1), 577: (1) → (0), 587: (1) → (2), 624: (1) → (0), 639: (2) → (1) |
| Node (13) | 52: (1) → (0), 55: (1) → (0), 84: (0) → (2), 88: (0) → (1), 147: (0) → (1), 148: (2) → (0), 293: (0) → (1), 363: (2) → (1), 396: (0) → (1), 758: (0) → (1), 782: 0 → 1 | 27: (2) → (0), 97: (0) → (1), 105: (0) → (1), 119: (0) → (1), 169: (1) → (0), 190: (0) → (1), 201: (1) → (0), 202: (0) → (1), 210: (1) → (0), 236: (2) → (3); 254: (1) → (0), 272: (1) → (0),  277: (1) → (0), 285: (2) → (1), 296: (0) → (1), 309: (0) → (1), 310: (0) → (1), 311: (0) → (1), 327: (1) → (0), 328: (0) → (1); 353: (1) → (2), 421: (0) → (1), 456: (1) → (0), 462: (1) → (0), 507: (0) → (1), 509: (0) → (1), 554: (1) → (0), 592: (0) → (1), 639: (1) → (0), 747: (0) → (1), 768: (2) → (1), 771: (0) → (1), 826: (1) → (0) | 377: (3) → (2), 379: (0) → (1), 516: (1) → (0), 701: (1) → (0), 784: (1) → (0) |
| *Tameryraptor* | 66: (0) → (1), 82: (1) → (0), 85: (0) → (1), 87: (1) → (0), 92: (1) → (0), 252: (1) → (0), 258: (0) → (1), 377: (2) → (1),  378: (0/1) → (2), 379: (1) → (2), 446: (1) → (0), 493: (0) → (2), 496: (1) → (0), 501: (1) → (2), 504: (0) → (1), 700: (1) → (0), 715: (1) → (0), 782: (1) → (2) |  | 414: (0) → (1), 421: (0) → (1), 507: (0) → (1), 712: (1) → (2), 785: (0) → (1) |
| Node (14) | 302: (0) → (1) | 95: (0) → (1), 256: (0) → (1), 301: (0) → (1), 370: (1) → (0), 401: (0) → (1), 423: (1) → (2), 501: (1) → (0), 614: (0) → (1), 712: (1) → (2), 726: (1) → (0) | 263: (1) → (0), 296: (0) → (1), 309: (0) → (1), 310: (0) → (1), 311: (0) → (1), 386: (0) → (1) |
| Node (15):  Carcharodontosaurinae | 72: (0) → (1), 154: (0) → (1) |  | 95: (0) → (1), 169: (1) → (0), 272: (1) → (0), 301: (0) → (1), 370: (1) → (0) |
| Node (16) | 158: (0) → (1) | 73: (1) → (0), 129: (1) → (0), 141: (0) → (1), 187: (1) → (0), 264: (1) → (0) | 27: (2) → (0) |
| Node (17):  Giganotosaurini | 39: (0) → (1), 43: (1) → (0), 265: (0) → (1), 269: (1) → (0) | 53: (0) → (1), 123: (0) → (1); 372: (0) → (1) | 97: (0) → (1), 202: (0) → (1), 254: (0) → (1), 277: (1) → (0), 290: (1) → (0), 376; (1) → (2), 456: (1) → (0), 680: (0) → (1), 747: (0) → (1) |
| Node (18) | 108: 1 → 0, 493: 0 → 1, 544: 1 → 0, 708: 3 → 1 | 27: (2) → (0), 116: (1) → (0), 208: (1) → (0); 258: (0) → (1), 260: (1) → (0), 421: (1) → (0), 507: (1) → (0), 554: (0) → (1), 576: (1) → (0), 640: (1) → (2), 662: (2) → (0), 680: (1) → (0), 726: (0) → (1), 801: (1) → (0) | 323: (0) → (1), 423: (1) → (2) |
| Node (19) | 320: (0) → (1), 741: (0) → (1), 744: (2) → (1) | 11: (1) → (0/1), 437: (0) → (1), 462: (0) → (1), 554: (1) → (0) | 208: (1) → (0), 328: (0) → (1), 374: (0) → (1), 507: (1) → (0), 657: (0) → (1), 726: (1) → (0) |
| Node (20):  Coelurosauria | 39: (0) → (1), 157: (0) → (1), 181: (0) → (2), 187: (1) → (0), 198: (1) → (0), 205: (0) → (1), 222: (2) → (1), 232: (0) → (1), 241: (2) → (1), 274: (1) → (0), 279: (1) → (0), 294: (0) → (1), 295: (2) → (1), 340: (0) → (1), 357: (0) → (2), 374: (0) → (1), 513: (0) → (3), 532: (0) → (1), 536: (1) → (0), 591: (0) → (1), 621: (0) → (1), 640: (0) → (1), 661: (0) → (1), 668: (1) → (0), 673: (0) → (2), 726: (1) → (0), 746: (0) → (1), 776: (0) → (1), 795: (1) → (0) | 203: (0/1/2) → (0/1), 213: (0) → (1), 286: (1) → (0), 305: (1) → (0), 322: (1) → (0), 483: (0) → (1), 486: (0) → (1), 491: (3/4) → (3), 524: (0) → (1), 583: (1) → (0), 619: (1) → (0), 678: (0) → (1), 697: (0) → (1) | 412: (1) → (0), 798: (0) → (1) |
| Node (21):  Tyrannosauroidea | 52: (2) → (1), 74: (0) → (1), 84: (0) → (2), 106: (0) → (1), 137: (0) → (1), 191: (0) → (2), 194: (0) → (2), 328: (0) → (1), 511: (1) → (0), 620: (1) → (2) | 4: (2) → (3); 38: (1) → (2); 67: (1) → (0), 79 (0) → (1), 88: (0) → (1), 124: (1) → (2 /3), 166: (0) → (1); 227: (0) → (1); 237: (0) → (1), 241: (1) → (0), 265: (0) → (1), 445: (0) → (1), 462: (0) → (1), 504: (0) → (1), 541: (0) → (1), 570: (0) → (1), 688: (0) → (1), 773: (0) → (1), 775: (0) → (1), 793: (0) → (1), 797: (0) → (1) | 323: (0) → (1), 183: (0) → (1) |
| Node (22) | 13: (1) → (0), 49: (0) → (1), 76: (0) → (1), 87: (1) → (0), 127: (0) → (1), 149: (0) → (1), 151: (0) → (1), 225: (0) → (1), 263: (1) → (2), 290: (1) → (0), 291: (0) → (1), 302: (0) → (1) | 44: (1) → (2/3), 80: (0) → (1), 161: (0) → (2), 190: (0) → (1/2); 204: (0) → (0/2/3); 224: (1) → (0), 252: (1) → (0), 264: (0) → (1), 363: (1) → (2), 409: (1) → (0), 504: (1) → (0) | 67: (1) → (0), 203: (0/1) → (0); 237: (0) → (1), 241: (1) → (0), 286: (0) → (1), 399: (1) → (2), 445: (0) → (1), 688: (0) → (1), 715: (0) → (1), 775: (0) → (1), 793: (0) → (1), 797: (0) → (1) |
| Node (23):  Megaraptora | 52: (1) → (2), 414: (1)  → (0), 574: (0)  → (1), 586: (0)  → (1), 641: (0)  → (1), 785: (1)  → (0) | 11: (1) → (0); 64: (1) → (2); 66: (1) → (0), 74: (1) → (0), 81: (0) → (1), 120: (0) → (1), 141: (0) → (1), 145: (1) → (0), 158: (0) → (1),  164: (0) → (1), 176: (0) → (1), 179: (0) → (1), 208: (0) → (2); 254: (1) → (0), 285: (2) → (0), 294: (1) → (0), 296: (0) → (1), 299: (0) → (1), 321: (1) → (0); 346: (0) → (1), 351: (1) → (0), 353: (1) → (0), 355: (0) → (1), 373: (0) → (1), 387: (1) → (0), 423: (1) → (2), 493: (0) → (2), 501: (1) → (2), 502: (1) → (2), 504: (0) → (1), 506: (0) → (1), 545: (0) → (1), 545: (0) → (1), 660: (0) → (1), 662: (2) → (0), 668: (0) → (1), 673: (2) → (0), 779: (1) → (0), 808: (1) → (0) | 239: (2) → (0, 1), 583: (1) → (0) |
