# Supplementary figures and images for: Re-evaluation of the Bahariya Formation carcharodontosaurid (Dinosauria: Theropoda) and its implications for allosauroid phylogeny
Source: PLoS One. 2025 Jan 14;20(1):e0311096. doi: 10.1371/journal.pone.0311096 (PMC11731741; doi:10.1371/journal.pone.0311096)

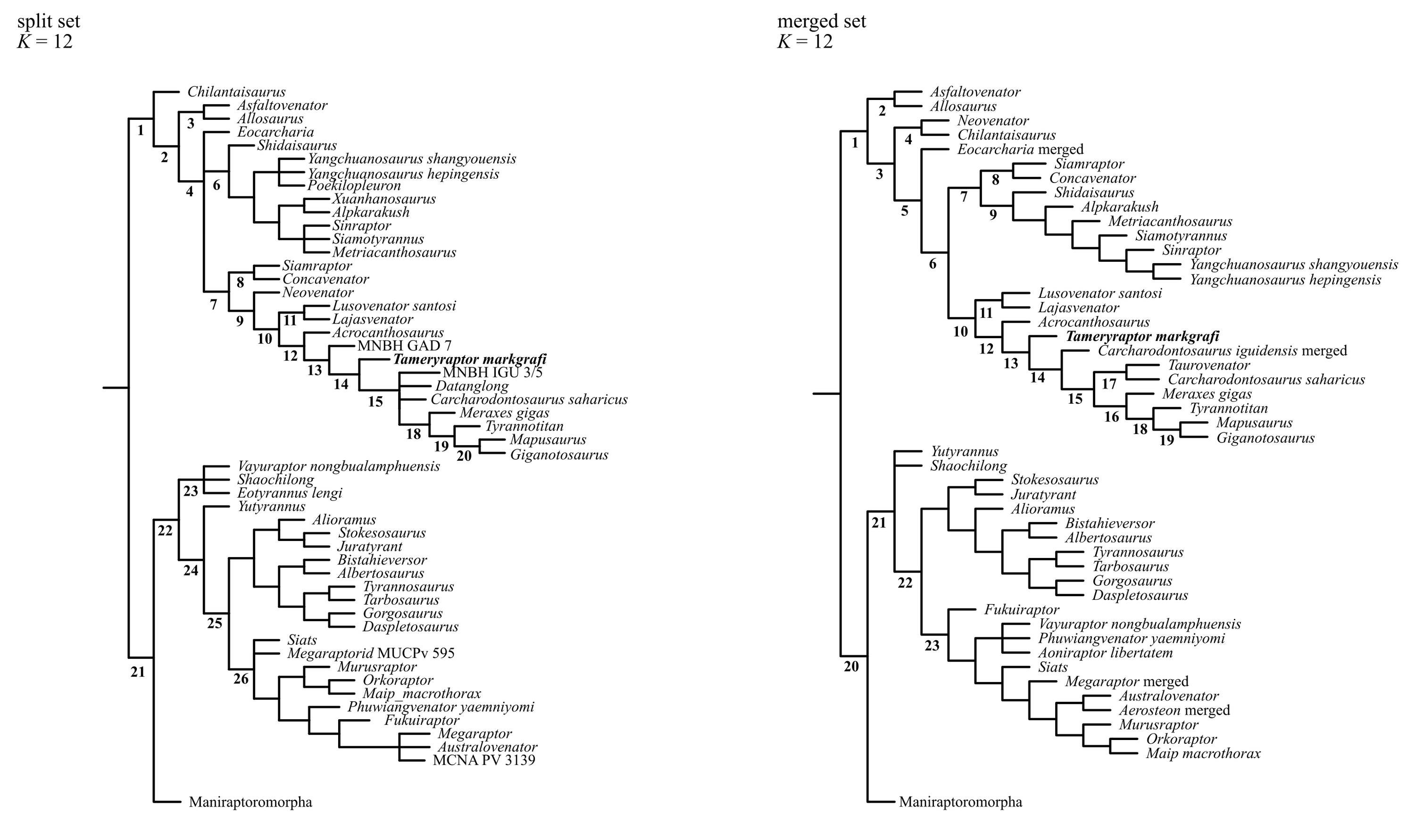

Supplement: S1 Fig — (TIF) [file pone.0311096.s006.tif]
