# Supplementary material for: Re-evaluation of the Bahariya Formation carcharodontosaurid (Dinosauria: Theropoda) and its implications for allosauroid phylogeny
Source: PLoS One. 2025 Jan 14;20(1):e0311096. doi: 10.1371/journal.pone.0311096 (PMC11731741; doi:10.1371/journal.pone.0311096)
Supplement: Attachment — Submitted filename: Response to reviewers.docx [file pone.0311096.s008.docx]

Reply to reviewers

We thank the reviewers for their critical and helpful comments, which have greatly helped to improve the manuscript. Most of the minor changes (especially those included in the annotated manuscript) were incorporated, and we will thus here offer mainly comments on some of the more severe criticisms and comments that we do not agree with.

Reviewer 1:

We will first reply to the reviewer’s main concern in general, and later to some of his or her sub-arguments in detail, as much of this is relevant for the question whether to base a new taxon on the now destroyed Egyptian material. For clarity, we will not copy all of the reviewers statements, but only those that we deem important for the main conclusions. Omitted parts are indicated by “…”

*Yet, I have several concerns about the taxonomic interpretation of the particular specimen lost in 1944, which is claimed by the authors to represent a new genus and species ("Alllissaurus markgrafi") and has been removed from the well-known taxon Carcharodontosaurus saharicus.
…
5) Brusatte and Sereno (2007) explicitly designated the Moroccan specimen as neotype of C. saharicus. This decision was based on the following arguments: 5a) the two teeth described by Depéret and Savornin (1925-27) likely pertain to different individuals; 5b) the diagnostic tooth characters originally cited are no longer diagnostic at the specific or generic level; and 5c) both Egyptian and Algerian specimens cannot be used as type material; 5d) the Moroccan specimen "is identical to both the original holotypic teeth and Stromer’s Egyptian material".
…*

*Despite the specimen being destroyed, the authors consider the published material sufficient for erecting a new genus and species, distinct from Carcharodontosaurus saharicus. Note that these statements challenge the statements 5c) and 5d) by Brusatte and Sereno (2007).*

*…*

*6) The Moroccan specimen does not belong to the same genus and/or species of the Egyptian specimen.
7) Even if the Egyptian specimen is lost, its description and the new photographs justify it as holotype for the formal erection of a genus and species .*

*Statement 7) is explicitly challenged by the International Code of Zoological Nomenclature (ICZN, 1999), Recommendation 73B: "An author should designate as holotype a specimen actually studied by him or her, not a specimen known to the author only from descriptions or illustrations in the literature". The Egyptian material, actually lost in 1944, should not be used to erect any new taxon.*

*…*

*The best way to preserve both Carcharodontosaurus and Carcharodontosauridae should be to designate the Egyptian specimen as neotype of C. saharicus because:*

In regards to statement 5):

Although the reviewer is correct with his/her historic review of the history of Carcharodontosaurus, we disagree with his/her later stated consequences from these findings. First, please note that Brusatte & Sereno (2007) explicitly state that the original syntypic teeth of *Megalosaurus saharicus* (named “holotype” in their paper) are not diagnostic, but they do not discuss anything about the potential diagnosabilty of the Egyptian material. Thus, the reviewers conclusion 5c (*both Egyptian and Algerian specimens cannot be used as type material*) is not based on an actual statement in this work, but was apparently deduced from the fact that Brusatte & Sereno chose the Moroccon skull (the only material still in existence) as neotype. However, this choice by Brusatte & Sereno is based on their assumption that the original type teeth, the Egyptian material and the Moroccon skull belong to the same taxon (the reviewer’s point 5d). The main point of our work is the interpretation that the Egyptian material represents a taxon distinct from the Moroccon skull. If our interpretation is correct (and we agree with Brusatte & Sereno that the original syntypic teeth are not diagnostic and probably lost), we are left with four possibilities

1) We ignore the Egyptian material, as it has been destroyed.

This is not really an option, as the material described by Stromer (1931, 1934, 1936) has been referred to repeatedly, especially in the more recent literature on Cretaceous Gondwanan theropods, and multiple authors have found Stromer’s descriptions and figures to be useful for anatomical and often even taxonomic comparisons and systematic/phylogenetic inferences. Thus, the interpretation that this material might represent a taxon different from other reference material currently referred to the same taxon is of interest to theropod workers, and simply ignoring it would perpetuate possible errors in the literature (e.g. basing phylogenetic codings on a mixture of the Moroccan skull and the material described by Stromer).

2) We change the neotype for Carcharodontosaurus saharicus from the Moroccon skull proposed by Brusatte & Sereno (2007) to the Egyptian specimen.

Although this seems to be a preferred option for the reviewer (see also below), we consider it to be a worse possibility than the one we adopted for several reasons. First, the designation of the Egyptian specimen as neotype for Carcharodontosaurus saharicus would only shift the focus of one of the main concerns of the reviewer, namely that a taxon should not be diagnosed on the basis of material that is no longer in existence. If we were to use the Egyptian material as neotype, we also would need to give a diagnosis for Carcharodontosaurus saharicus, based on Stromer’s descriptions and the existing illustrations and figures – the same as for the erection of a new taxon.

Second, the designation of the Egyptian material as neotype would directly violate two of the qualifying conditions for neotype designation laid out by the ICZN (articles 75.3.6 and 75.3.7). Article 73.3.6 states that a requirement needed is “evidence that the neotype came as nearly as practicable from the original type locality … and, where relevant, from the same geological horizon or host species as the original name-bearing type”. The Egyptian specimen came from a locality approximately 2800 km distant from the original type locality of Megalosaurus saharicus at Timimoun in Algeria, and the beds there were originally assigned to the Albian by Depéret & Savognin (1925, 1927), whereas the Baharyia Formation has since Stromer’s times been considered to be Cenomanian in age. In contrast, the locality in the Douira Formation from where the skull that was made the neotype by Brusatte & Sereno (2007) was collected, is only about 470 km away from Timimoun and is placed in sediments that are generally (at least roughly) correlated with the rocks outcropping at the type locality (see Lapparent 1960; Mateer et al. 1992; Ibrahim et al. 2020); the interpretation that all of these units are Cenomanian in age (as the Baharyia Formation) is actually based on faunal comparisons with the Baharyia-Formation – which we argue to be problematic in the discussion of our paper. Article 75.3.7 states that “…the neotype is, or immediately upon publication has become, the property of a recognized scientific or educational institution…”. As the material from Egypt is lost, it cannot be placed in a recognized scientific or educational institution – thus precluding it from becoming a neotype on this basis alone (unless, again, one considers the descriptions and illustrations by Stromer and the photographs in the BSPG and UT archives as a valid type – in which case there is no reason why not to base a new taxon on these).

Third, the qualifying condition of article 73.3.5 “evidence that the neotype is consistent with what is known of the former name-bearing type from the original description and from other sources” is also problematic for the Egyptian material. In this context, it might be important to point out that Stromer (1934: p. 62) designated the smaller tooth described by Depéret & Savornin (1925, 1927) as “type” of Carcharodontosaurus saharicus, which we consider to be a valid lectotype designation according to article 74.5 of the ICZN. Stromer (1931: p. 9) compares this tooth with a tooth that was found isolated in the locality of his specimen, which he thought to represent the same taxon, namely Carcharodontosaurus saharicus. However, he also pointed out that this tooth differed in the stronger recurvature from the teeth found in situ in the maxilla of the specimen he described. Given that the general features of carcharodontosaurid teeth are now known to be not diagnostic on species or even genus level, it cannot be ruled out that the isolated tooth found with Stromer’s specimen came from another carcharodontosaurid taxon scavenging on the carcass. Thus, there is a – admittedly small and of currently unknown taxonomic significance – difference in curvature between the type tooth of Carcharodontosaurus saharicus and the teeth found in situ in the Egyptian specimen. In contrast, the smaller tooth described and figured by Depéret & Savornin (1927: pl. 13, fig, 1) is in complete agreement with teeth found in the Moroccan skull.

Fourth, as we consider the neotype designation of the Moroccan specimen by Brusatte & Sereno (2007) to be valid (see below), a new neotype designation would directly violate article 75.4 of the ICZN: “The first neotype designation published for a nominal species-group taxon in accordance with the provisions of this Article is valid and no subsequent designation, except one made by the Commission under the plenary power…, has any validity”.

Thus, we reject this option for all the reasons outlined above.

3) We leave the Moroccon skull as neotype for Carcharodontosaurus saharicus, simply state that we think that the Egyptian material represents a different taxon, but do not propose a new binomen for it.

This would be an option, but we consider it less good than the one we chose. If one considers Stromer’s descritpions (which are quite detailed for its time) and his illustrations to be accurate, the material from Egypt is clearly diagnosable. That Stromer’s descriptions and figures are accurate is supported by his descriptions of other materials that are still around and can be compared with the descriptions, such as the type specimens of Lybicosuchus and Aegyptosuchus (both kept in the BSPG collections), and by the newly found photographs of the materials that show many of the features that he describes and generally agree with the drawings of his publication. Thus, we would be left with a clearly diagnosable new taxon without a formal taxonomic name. This we consider problematic for two rather practical reasons. First, a named taxon receives considerably more attention than an unnamed taxon, and as the Egyptian material currently represents the only diagnosable carcharodontosaur from Africa east of the inner-Saharian seaway, it is an important reference for Cretaceous Gondwanan theropod faunas. Second, if one considers the material diagnosable (for which we, hopefully, make a strong case), it will be only a matter of time until the taxon will be named, if not by us then by subsequent authors who might not be aware or not agree with the reasons not to name a new taxon on diagnosable material, even if it is no longer around.

4) We leave the Moroccon skull as neotype for Carcharodontosaurus saharicus and propose a new taxon for the (now destroyed) Egyptian material.

This is the option we chose, for all the reasons explained above. As the neotype designation by Brusatte & Serene has to be regarded as valid (see below) and we regard the Egyptian material as clearly distinct from other carcharodontosaurids and also diagnostic, this seems to be the best option.

*Statement 7) is explicitly challenged by the International Code of Zoological Nomenclature (ICZN, 1999), Recommendation 73B: "An author should designate as holotype a specimen actually studied by him or her, not a specimen known to the author only from descriptions or illustrations in the literature". The Egyptian material, actually lost in 1944, should not be used to erect any new taxon.
The ICZN recommendation must be followed for a series of epistemological reasons. The first is the non-reproducibility of the observations reported in support of the new species: the irreversible destruction of the specimen prevents any objective evaluation of the diagnostic features introduced. Accordingly, any emendation of the diagnosis cannot be falsified. The second is the non-testable nature of a series of ambiguous interpretations of the published photos used by the authors to suggest some features not explicitly reported by Stromer (1931).*

As noted by the reviewer himself, the ICZN statement is a recommendation, not a binding article. Of course, it is always preferable to base taxa on material that can be studied firsthand and that is available for subsequent study. However, as this material has been destroyed a very long time ago, this is obviously not an option in this case. Nevertheless, regardless of the recommendation cited by the reviewer, ICZN code 73.1.4 generally allows description of a new nominal taxon based on previous descriptions and illustrations, and there are numerous examples both in palaeontology and biology where this has been done. Thus, in dinosaurs, for example, the sauropod taxon *Nopcsaspondylus alarconensis* Apesteguía, 2007, was based on a single sauropod vertebra described and figured in three views by Nopcsa (1902) and was apparently lost long before the erection of a new taxon on it. Similarily, Amphicoelias fragillimus Cope, 1878, was described on a single neural arch for which only a field sketch existed, and Carpenter (2018) even changed the generic name for this taxon to Maraapunisaurus based on this illustration alone.

More commonly, new species are described on specimens that might still have been studied by the authors but were not collected or lost prior to the description of a new species. Thus, for example, the Devonian lungfish Apatorhynchus opistheretmus Friedman and Daeschler, 2006, is based on a partial skull roof that was never collected, so that only photographs and a cast exist. More recently, Brum et al. (2021) described the unenlagiine theropod Ypupiara lopai on the basis of a partial maxilla that was destroyed previously in the fire that destroyed the Museu Nacional in Rio de Janeiro in 2018. Furthermore, in some types of fossils, for example vertebrate tracks, it is common practice that the actual specimens are not collected, but new ichnotaxa are described on the basis of photographs, photogrammetry or casts.

In all of these cases, the epistemological reason raised by the reviewer, that the lack of the actual specimen precludes verification of the finds, would also apply. Nevertheless, it is generally considered to be acceptable that the documentation presented in the papers is sufficient to characterize the new taxon. In our case, the anatomical interpretations and diagnostic features are all based on the description by Stromer, the detailed drawings included in his publications, and the newly found photographs, and thus, all of our statements can be verified or contested on the basis of the same data. We have been careful in our interpretations of the available data and have always indicated uncertainty whenever it is present in our descriptions. Whenever this uncertainty is not stated, our observations can be clearly verified from the descriptions, figures or images of the specimen, or a combination of either sources (see comments on features contested by the reviewer below). Furthermore, the ICZN gives recommendations for cases where the designation of a new taxon based on description and illustrations of a non-existing type, which we followed:

*“****Recommendation 73G. Specific reasons for designation of an unpreserved specimen as the name-bearing type.*** *An author should provide detailed reasoning why at least one preserved specimen, whether a complete individual organism or a part of such an individual, was not used as the name-bearing type for the new taxon and why the formal naming of the taxon is needed at a point in time when no preserved name-bearing type will be available.”*

- As can be shown from Stromer’s descriptions and illustrations, the Egyptian carcharodontosaurid is distinct from any other known carcharodontosaur species. As this was the only specimen of its specific taxon ever recovered, no preserved specimen is available to select as a name-bearing type.

*“****Recommendation 73H. Assertion of due diligence.*** *When establishing a new species-group taxon without a preserved name-bearing type, steps taken by an author to capture and preserve a physical specimen of the new taxon and/or locate an existing preserved specimen in natural history collections should be recounted.”*

- It has been known for a long time that the original specimen was destroyed in 1944 during WW 2, and ever since the destruction of the original material, expeditions in the Bahariya Oasis were unable to retrieve another specimen for this taxon (see e.g. Nothdurft et al. 2002: “The Lost Dinosaurs of Egypt”)
- We have thoroughly searched both the archives of the BSPG and the UAT to find as much information on this now lost specimen as possible. As a result, we were able to recover two photographs of the specimen

***“Recommendation 73I. Consultation with specialists.*** *Before the designation of an unpreserved specimen as a name-bearing type, an author should consult with specialists in the group in question.”*

- The first author of the paper has also worked on theropods since his bachelors degree in 2018 and has been part of the Mesozoic Tetrapod Working Group, developing a large phylogenetic dataset for theropods.
- The remaining two authors of the paper (EC and OWMR) are experts in theropod anatomy, taxonomy and systematics, and specifically qualified in this case due to their many years of experience working on theropods in general and carcharodontosaurids in particular.

*“****Recommendation 73J. Comprehensive iconography and measurements.*** *When establishing a new species-group taxon without a preserved name-bearing type, the author should provide extensive documentation (e.g., multiple original high-resolution images, DNA sequences, etc.) of potentially diagnostic characters as completely as possible.”*

- Multiple detailed drawings of the specimens exist and they are supplemented by the two newly discovered and previously unpublished photographs. Combined with elements of Stromers original detailed descriptions, we were able to provide 13 pages of anatomical description and a table spanning two and a half pages of measurements to accompany the description of our new species. The combination of characters present in the Egyptian charcharodontosaurid is unique among the group and further accompanied by multiple autapomorphies, such as the internal nasal suture being inclined ventromedially (both described by Stromer 1931: p 6 and visible in our Fig. 5), dorsally vaulted frontals (described by Stromer and visible in the photograph of the specimen, as indicated in our Figure 6 C) and anterior caudal vertebrae with foramina invading the spinoprezygapophyseal fossa from the lateral side of the neural arch (both described by Stromer 1931: p 12 and visible in our Fig. 9 D,F)

Regarding further critiques on reproducibility falsifiability Reviewer 1 also noted the following:

*For example, the authors wrote:
"An expansion of the narial fossa to the nasal in the form of a supranarial fossa, as it is present in Acrocanthosaurus, Concavenator, Carcharodontosaurus and Meraxes [11,18,43,44] is not clearly visible in the photo of SNSB-BSPG 1922 X 46, but Stromer [1] described the area of the anterior end of the nasal lateral to the median premaxillary process as slightly concave. However, a clearly defined rim of such a fossa, as it is present in Carcharodontosaurus ([18]: Fig 134A) is not at all indicated in Stromers figures, nor visible on the photograph."
The authors implicitly remark that the development of key features is ambiguous once the literature is compared with the photos/illustration. What should be considered the valid source? In this case, the narial fossa is described by Stromer (1931) as a slight concavity, yet such feature is not illustrated in the figures/photos.*

This seems to be a misunderstanding of the structures meant here. Our observations and statements do not contradict Stromers descriptions in this case. We do not judge the presence of a supranarial fossa in the Egyptian carcharodontosaurid as such (Stromer did mention a slight concavity being present in the location where such a feature would be expected), but we state that a clearly defined rim demarcating such as fossa, as it is present in Acrocanthosaurus, Concavenator, Carcharodontosaurus and Meraxes (see e.g. Eddy & Clarke 2011 Figure 3 for an example for how this rim is developed if it is present), cannot be seen in either Stromer’s drawings, nor in the photograph of the element. While the development of the fossa itself is thus indeed ambiguous, as stated by Reviewer 2 (since such a subtle structure is not visible in any of the drawings or pictures), the existence of a strong rim demarcating a fossa seems unlikely, as the drawings are usually reliable in respect to such clearly defined structures, and no trace of it is visible in the photograph that otherwise clearly shows structures of comparable size and clarity (e.g. the ornamentation of the maxilla). Thus, this would be a character differentiating the Egyptian carcharodontosaur from the aforementioned taxa.

*The authors also wrote:
"The antorbital fossa extends onto the lateral surface of the nasals, which represents an allosauroid synapomorphy [7,47], and is clearly visible in lateral view on the nasals of SNSBBSPG 1922 X 46. This is visible in the photograph of the specimen and also indicated in Stromer’s reconstruction of the skull (Figs 2 and 4B, D)" Contrary to the authors, I hardly differentiate an antorbital fossa of the nasal in the photo of the Egyptian specimen from the homologous part in the Moroccan specimen. The description by Stromer (1931) is ambiguous on such feature, so in this case we have to trust the interpretation of the text and figures by the authors.. …... The authors might be right in their interpretation of the images, but without direct observation of the specimen, there is no way for eventually falsifying their statement. If they are wrong, such error is fixed in their diagnosis of the taxon and could not be corrected by any independent observation. The drawings by Stromer do not match all details claimed from the photos, so what of the two images should we trust? Some features claimed by the authors could be non-biological in origin, due to taphonomy or damage, or even just photographic artifacts. If the authors have "over-interpreted" some features in the only available images of the now destroyed Egyptian specimen, nobody could falsify them. Thus, the diagnosis of "Alllissaurus markgrafi" is epistemologically problematic and cannot be considered a proper scientific statement.*

Contrary to the statement of reviewer 2, this feature is indeed clearly visible in the photograph of the specimen (Fig. 2), and also clearly indicated in Stromers 1936 sketch of the specimen (Stromer 1936: p: 66), as already pointed out by Rauhut (1995). Furthermore, our observations are also indirectly supported by Stromers comparisons of the specimen. While he did not explicitly state the presence of the antorbital fossa in his 1931 description, he did directly compare the nasals of the Egyptian carcharodontosaurid with the nasals of Allosaurus (Stromer 1931: p 6 “In der Gesamtform sind nur die Nasalia von Antrodemus (Gilmore 20, p. 18, Fig. 9) vergleichbar.”). He furthermore noted the presence of pneumatic openings on the lateral side of the nasals anterior to the lacrimal articulation (i.e. in the antorbital fossa of the nasals of Allosaurus), as a difference to the Egyptian carcharodontosaurid, meaning he directly compared this specific anatomical region in the two taxa (See Stromer 1931 p:6 Aber diese sind bei dem stattlichen Individuum Nr. 4734 ….. am Lateralrande mit einem rückragenden Fortsatze für das Lacrymale und davor endlich mit Öffnungen in innere Hohlräume.”). In the same, arguably somewhat convoluted sentence, he also noted differences in rugosities of both nasalia, as well as the differences in the nasal process (anteriorly split vs undivided), maxillary process (long vs short) and lacrimal articulation in both taxa. It would be uncharacteristic for Stromers descriptions to not mention a marked difference such as the absence or presence of the antorbital fossa on the lateral side of the nasals, if all of these other differences are also noted.

One aspect that might have led reviewer 1 to doubt our interpretation is that, in the pdf document provided for revision, the antorbital fossa is indeed hardly visible, due to the lower quality of the images. However, this structure is clearly visible in the original, high quality figures that were uploaded with the submission.

*Stated in a simpler way: if the authors are wrong in some interpretations, how could them be falsified without an available specimen? A couple of old black-and-white photos alone (one of which clearly was not meant to illustrate anatomical details but just the whole mounted material) is not sufficient evidence that all features listed by the authors as diagnostic are actually natural apomorphies.*

As already noted above, we were rather cautious in our interpretations, and acknowledge the cases where uncertainty about the morphology exists. Furthermore, apart from the features picked out by the reviewer, we list a number of further differences that were clearly described and are visible in the available illustrations that distinguish the Egyptian specimen from the Moroccan skull, and, in their entirety, represent a unique character combination for the material described by Stromer (e.g. presence of a nasal horn, angled internasal suture, vaulted frontals), so the distinctiveness and diagnosability of the Egyptian carcharodontosaurid do not hinge on the characters contested by the reviewer.

*Other features of the Egyptian specimen, even if genuine, could not be significant from a taxonomic perspective. For example, the "nasal horn" and the more extensive maxillary subcutaneous ornamentation (claimed to be absent or less developed in the Moroccan specimen) could be ontogeny-related and/or sexually-dimorphic features widespread among the carcharodontosaurids once a richer sample is available.*

This is undoubtedly true, but is not a statement that has anything to do with whether the specimen still exists or not. There are numerous instances in which cranial crests, horns or bosses have been used for diagnoses of theropod taxa that are known from single individuals or a very limited sample size (e.g. Ceratosaurus, Carnotaurus, Monolophosaurus, Guanlong, Proceratosaurus, Majungasaurus, Caihong, several species of oviraptorosaurs), for which the same argument could be made. Until further specimens become available, it will be always impossible to tell if such structures might be sexually dimorphic or ontogenetically variable (note, though, that many other carcharodontosaurids, for which no such ornaments are known, are based on at least subadult specimens, making an ontogenetic variation rather unlikely).

*Statement 6) is an even more complex issue. The authors apparently seem unaware of the full implications of their main hypothesis: if we accept that the lost Egyptian specimen could be used as valid type of a taxon, and also endorse the taxonomic distinction of the Egyptian and Moroccan specimens, this has relevant nomenclatural implications:
First, if the Moroccan specimen does not belong to the same taxon as the Egyptian specimen, then the rationale for erecting a neotype of C. saharicus by Brusatte and Sereno (2007) from the former specimen is no more valid. In fact, the neotype was introduced under the assumption that all specimens mentioned belong to a single taxon. If the two specimens belong to different taxa, one cannot be used as the neotype which replaces the destruction of the other: the neotype sensu Brusatte and Sereno (2007) is thus invalidated!*

We cannot follow the argumentation of the reviewer here. For a neotype designation, it is irrelevant if all of the specimens previously referred to this taxon indeed represent the same taxon or not, what is relevant is that the holotype (or lectotype or syntype; in this case the Algerian teeth) is lost and needs to be replaced by a suitable specimen for the sake of nomenclatorial stability. The requirements for this are clearly stated in article 75.3 of the ICZN and have been followed by Brusatte & Sereno (2007). Most importantly, these authors discuss at length why they consider the syntype teeth (Brusatte & Sereno were obviously not aware of Stromer’s lectotype designation) of Carcharodontosaurus saharicus to be lost, and that they, on the basis of our current knowledge, are considered as undiagnostic, from which a necessity of a neotype designation is obvious (see also above for other requirements such as that the neotype should be from a locality as close as possible to the original locality). Please note also that Sereno et al. (1996: 987) explicitly stated that the referral of the Moroccon skull to Carcharodontosaurus saharicus was based on the fact that the teeth were identical to the ones described by Depéret & Savornin (1925, 1927), i.e. the syntype teeth of this species. The only statement of Brusatte & Sereno (2007: 904) to that respect is the sentence cited by the reviewer (“More recently, Sereno and colleagues (1996) described a well preserved skull with teeth from Morocco that is identical to both the original holotypic teeth and Stromer’s Egyptian material”). Here, the authors again state that they consider the teeth of the Moroccon skull to be identical to the type teeth. That they also (in our view erroneously) consider the Egyptian material to be identical to the Moroccon skull is of no consequence for the neotype designation, as the Egyptian material is a referred specimen. This is clearly stated by Stromer (1931: 19), who considered his material to belong to the same taxon as the teeth described by Depéret & Savornin and thus considered it a referred specimen (he also cites the species as “Carcharodontosaurus saharicus (Deperet et Savornin)” in his 1934 and 1936 papers). Note that even in cases where a nominal taxon is founded on material later found to represent more than a single taxon, the availability of the name is not affected (ICZN article 17).

*Furthermore, if the lost Egyptian specimen is now considered valid as holotype of "Alllissaurus", then it means that it has always been the valid type for a carcharodontosaurid taxon lacking a neotype: in that case, it has always been the most appropriate neotype of Carcharodontosaurus in spite of the Moroccan specimen, because Stromer (1931) used it to erect and diagnose that genus. Accordingly, the genus name "Alllissaurus" is automatically a junior synonym of the genus name Carcharodontosaurus.*

As outlined above, although Stromer does propose a new genus name, he explicitly did so for it to include the species *Megalosaurus saharicus*. Concerning the rules of the ICZN, it therefore does not really matter if Stromer’s specimen was an “appropriate neotype” – it was never designated as such, and, as pointed out above, considered to be a referred specimen in its original description. That he changed the genus name is of no concern for the neotype designation of the species. Indeed, article 68.3 of the ICZN states that “When an author establishes a new nominal genus-group taxon for a single taxonomic species and denotes that species by an available name, the nominal species so named is the type species.” Stromer (1931: 19) creates the new generic name and explicitly includes the available nominal species Megalosaurus saharicus in this genus, as Carcharodontosaurus saharicus. As this was the only species included in the genus, it automatically became the type species of this genus, as by article 68.3 cited above. Thus, the valid neotype designation of the Moroccon skull for Carcharodontosaurus saharicus fixes both the species and the genus name to this specimen.

*The authors might reply that Carcharodontosaurus is now anchored to the Moroccan specimen, but such nomenclatural act was historically based and conditioned to a taxon sample the authors here have just demonstrated being wrong. They wrote: "In the interest of preserving the widely used genus Carcharodontosaurus and family Carcharodontosauridae, we concur with the designation of SGM-Din 1 as the neotype of Carcharodontosaurus saharicus sensu Brusatte and Sereno". Yet, such statement is contradicted by the main argument of their manuscript, because the authors have just (implicitly) demonstrated that the rationale for designating SGM-Din 1 [the Moroccan specimen] as valid neotype for Carcharodontosaurus is no more valid, because the Moroccan and Egyptian specimens do not belong to the same genus.*

As outlined in detail above, the argumentation of the reviewer here has no base in the ICZN, and is contradicted by the validity of the neotype designation of the Moroccon skull by Brusatte & Sereno (2007).

*The best way to preserve both Carcharodontosaurus and Carcharodontosauridae should be to designate the Egyptian specimen as neotype of C. saharicus because:
1) The Egyptian specimen - assumed by the authors as a potentially valid type specimen for a taxon - has been the explicit source for the diagnosis of Carcharodontosaurus since the erection of the genus in 1931, whereas the Moroccan specimen was introduced only in 1996 and has been (arbitrarily) considered the neotype of the same taxon of the Egyptian specimen only in 2007 to replace the destroyed material.
2) All literature prior to 2007 assumed the Egyptian specimen as the main source of Carcharodontosaurus diagnosis (and, so far, it keeps being the only available source for all postcranial features of this taxon): even the referral of the Moroccan specimen to that taxon (regardless of it being right or wrong) was merely a consequence of comparing it with the Egyptian material. Removing the latter from Carcharodontosaurus is everything but an act "preserving" Carcharodontosaurus stability and consistency.*

Even if we would not consider the neotype designation by Brusatte & Sereno (2007) as valid and thus any subsequent change of neotype to violate ICZN rules, the argument of the reviewer here is also questionable. As summarized by Rauhut (1995), Carcharodontosaurus saharicus did not receive much attention in the literature prior to 1995, other than in overview articles and books, such as Huene’s (1956) “Paläontologie und Phylogenie der niederen Tetrapoden”, Romer’s (1966) “Vertebrate Paleontology”, or the 1990 compendium “The Dinosauria”. Only after the discovery of the Moroccan skull (1995, published by Sereno et al. 1996) and the recognition that other Gondwanan theropods belonged to the same clade, the name Carcharodontosaurus and the Carcharodontosauridae became more generally used, but, from that time on, always in reference to both Stromer’s material and the skull described by Sereno et al. (1996).

*There is another reason which justifies the removal of the Moroccan specimen from C. saharicus instead of the Egyptian one: as stated above, according to the ICZN Recommendation 73B, "an author should designate as holotype a specimen actually studied by him or her, not a specimen known to the author only from descriptions or illustrations in the literature". Given that, of the two specimens, only the Moroccan one is currently housed in a public institution, only the latter could be eventually studied by a researcher, and so it is the only one of the two which is available if a second taxon is eventually split from the Carcharodontosaurus hypodigm*.

As outlined above, removal of the Moroccan specimen would violate ICZN rules, but we also fail to see why it would be preferable to base the diagnosis of an existing species on a specimen that is no longer available over the diagnosis of a new species on said specimen, as the problems and implications are the same in both cases.

*A fourth reason for challenging the exclusion of the Egyptian specimen from Carcharodontosaurus is that the support of the phylogenetic results is weak. As shown in detail by the authors, when compared to the shortest scenario found by the analyses, only two additional steps are necessary for producing a Carcharodontosaurus saharicus node including both Egyptian and Moroccan specimens, and only three additional steps for reconstructing a node with all Carcharodontosaurus specimens in the monophyletic genus (both C. saharicus and C. iguidensis material). I replicated the analyses and confirm the results. Such small step differences are not statistically significant once evaluated with the Templeton test: we cannot exclude that they are merely due to lack of overlapping data and/or even a few coding artifacts (eventually biased by some miscoding due to misinterpretation of the published source). This means that the monophyly of Carcharodontosaurus keeps being a potentially valid topology. I would not consider the phylogenetic results sufficiently robust for supporting the splitting of the Egyptian specimen from Carcharodontosaurus. The radical taxonomic revision of a key historical specimen based on such weak results is thus poorly supported and should be avoided.*

This represents an often made misconception of the relation between taxonomy and phylogeny. Taxonomy seeks to differentiate taxa from one another, whereas phylogeny searches for their evolutionary relationships. Thus, two taxa might have the exact same phylogenetic codings, but still represent different taxa. As usually autapomorphic characters are not included in phylogenetic analysis of morphological data, it will actually often be the case that sister taxa are not distinguished by phylogenetic characters – but you would usually not argue that sister taxa therefore automatically become to be the same species. Thus, the question here is if the differences noted by us between the Moroccan and Egyptian specimens warrant differentiation on the species and genus level. That the two specimens come out in different positions in our phylogenetic analysis, albeit weakly supported, is further evidence that they do.

Furthermore, given the very fragmentary fossil record of carcharodontosaurids, combined with the very limited overlap between the North African specimens, the small number of additional steps is not an unexpected result. Similarly constraining other carcharodontosaurids into a sister group relation with one another requires a similarly small number of additional steps. It only takes one more step to constrain the Egyptian carcharodontosaurid into a sister group relation with the South American Meraxes (resulting score 6489), which is even fewer than the “saharicus” constrain (+2 steps). Following this reasoning, it would be more adequate to synonymize these Egyptian and South American taxa. Likewise, forcing the Egyptian taxon and Datanglong into a sister taxon relationship also requires a single additional step, as does forcing Carcharodontosaurus saharicus (the Moroccan skull) into a sister-taxon relationship with Tyrannotitan, or Carcharodontosaurus iguidensis into a sister-group relationship with Meraxes. However, all of these taxa are usually regarded as separate species or even genera and sometimes separated by continents and millions of years from oneanother.

**In summary, following the ICZN rules, we consider the Moroccan skull to have been validly designated as neotype of *Carcharodontosaurus saharicus*. Therefore, a new name is needed for the (unfortunately lost) Egyptian specimen of carcharodontosaurid to distinguish it from this species and underline its importance for our understanding of carcharodontosaurid evolution and biogeography. In the light of the reviewer’s comments, we added further discussions of our reasons for distinguishing the two taxa in the respective section of the manuscript (in accordance with recommendation of ICZN recommendation 73G). We thank the reviewer for his comments that helped to sharpen up this crucial section of the manuscript!**

*Note that the taxonomic section of the manuscript lacks any record of ZooBank registrations, making any nomenclatural act - regardless of their substantial validity - as formally invalid (e.g., if the manuscript is accepted in the current version, "Alllissaurus markgrafi" is a nomen nudum).*

We noted the necessity of adding a zoobank registration in the manuscript, but refrained from registering the new taxon before the paper is accepted. The necessary link will thus be added at a later stage.

Reviewer 2:

*If the information in p. 10 ¶3 could be summarized in a table (in particular, the OTU used in each “separate” and “merged” configuration), that would be appreciated.*

As suggested, we added Table 1, summarizing the differences in Merged and Split datasets.

*……  they themselves repeat an invalid referral by taking some of Stromer’s writings at their word. Stromer’s (1931, 1934) comparisons of various African dinosaurs to “Dryptosaurus” is not to the New Jersey tyrannosauroid Dryptosaurus aquilunguis; rather, it is to the dinosaur universally accepted today as Albertosaurus sarcophagus (but which was in the late 19th and early 20th Century referred to the tooth taxon Laelaps/Dryptosaurus incrassatus. See Stromer’s footnote on the bottom of page 56 in his 1934 paper: “Nach PARKS (1928, S. 5-7) ist Dryptosaurus LAMBE (1904, S. 20, Raf. VI, Fig. 6) warhscheinlich mit Albertosaurus sarcophagus OSBORN identisch.”
And his reference in the 1931 paper to the North American Dryptosaurus refer to Lambe’s 1904 paper of the skulls which became the type and coptype of Albertosaurus sarcophagus in Osborn 1905. Thus, in this paper of confusing theropod taxonomy, some proper clarification should be made that Stromer’s use of “Dryptosaurus” is actually Albertosaurus. (See p. 20 ¶3)*

The reviewer is certainly right here about the confusion of several taxa that are currently regarded separate species of tyrannosaurids in Stromer’s writing on Carcharodontosaurus. However, our only reference to Dryptosaurus refers to the fact that Depéret & Savornin (1927) referred the teeth they originally described as Megalosaurus saharicus to the genus Dryptosaurus, apparently largely on stratigraphic reasons. Thus, Stromer’s comparisons of other material (in his 1934 paper this concerns a metatarsal, which we do not consider here, as it has no overlap with the original specimen) with Dryptosaurus (=Albertosaurus) is of no concern for our paper.

*An aside: I appreciate the evocative name Alllissaurus (marauder reptile), but note in advance that this name is destined to be consistently misspelled (triple “l”s are rare), and will almost assuredly be autocorrected (either in word processors or web searches) to “Allosaurus”! This isn’t a reason not to use the name by any means, but simply looking ahead*

We appreciate this input on our suggested name for the taxon and have changed the genus name accordingly to Tameryraptor.

*p. 35, ¶3 The authors should make clear the definition of “Allosauroidea” they are using. I am assuming (given the authorship of this manuscript) they follow Rahut and Pol (2019), namely the most inclusive clade containing Allosaurus fragilis but not Megalosaurus bucklandii or Neornithes. Some previous node-based definitions would be potentially synonymous with Allosauria or the new Carcharodontosauriformes depending on the configuration of the allosaurs they found.*

We added an indication of which definition of Allosauroidea is followed in this paper after the clade is first mentioned.

*Cladograms: Note that Alpkarakush has yet to be published; please make certain of the timing of the publication of this paper vs. the Alpkarakush paper so that the authors do not trump themselves*

The Alpkarakush manuscript has spent an unexpectedly long time in review, but has now been accepted for publication; thus it is cited as “forthcoming” in our manuscript. Of course, the publication date is off our hands. We hope that the two manuscript will be published in due order.
