# Supplementary material for: Re-evaluation of the Bahariya Formation carcharodontosaurid (Dinosauria: Theropoda) and its implications for allosauroid phylogeny
Source: PLoS One. 2025 Jan 14;20(1):e0311096. doi: 10.1371/journal.pone.0311096 (PMC11731741; doi:10.1371/journal.pone.0311096)
Supplement: Attachment — Submitted filename: Response to reviewers.docx [file pone.0311096.s009.docx]

**Reply to reviewers**

We thank the reviewers for their additional revisions and critiques. In the following, we will once more address the criticism of the reviewers separately.

**Reviewer 1:**

We thank reviewer 1 for the helpful reviews and we appreciate the concerns brought forward about possible epistemological issues that may arise from naming a new taxon. We have been debating this issue ourselves for a while and yet remain with our conclusion, that naming the Egyptian carcharodontosaur is a preferable solution over leaving a clearly diagnostic and scientifically significant taxon unnamed.

The main concern of reviewer one *“*how could we falsify Stromer's words in the case they (the words) were wrong*?”* is valid, but has been thoroughly considered by us. In choosing the diagnostic features of our taxon, we did not use any features based solely on Stromers statements alone (Even though these are generally very accurate, as can be verified by crossreferencing Stromers publications with still existing material housed in the BSPG, like for example *Lybicosuchus*). For our taxon diagnosis we used only characters that are either

A) Directly visible on the picture of the specimen and are further supported by Stromers descriptions and/or illustrations of the specimen
B) highly unusual features that would be hard to misinterpret and are clearly described by Stromer and clearly visible in the illustrations of the specimen

For a overview of this see the following Table:

| Character used in diagnosis | Visible in Photograph | Visible in Illustrations | Described by Stromer |
| --- | --- | --- | --- |
| *antorbital fossa visible on nasals in lateral view* | UAT 678/20 | Stromer 1936, p: 66 Abb. 9 C | Stromer 1931 p. 6 |
| *small nasal horn on the medial rim of the nasals** | UAT 678/20 | Stromer 1931, pl. 1 Fig. 7a, b  & Stromer 1936, p: 66 Fig. 9 C | Stromer 1931 p. 6 |
| *Maxilla with well-developed antorbital ridge* | UAT 678/20 | Stromer 1931, pl. 1 Fig. 6a  & Stromer 1936, p: 66 Abb. 9 C | Stromer 1931 p. 7 |
| *maxillary teeth highly symmetrical anteroposteriorly* | Our Fig. 3 | Stromer 1931, pl. 1 Figs. 2; 6b | Stromer 1931 p. 7  Stromer 1934b (On the teeth of compsognathus) |
| *prefrontal facet on frontal not expanded* | UAT 678/20 | Stromer 1931, pl. 1 Fig. 4a | / |
| *frontals vaulted dorsally** | UAT 678/20 | Stromer 1931, pl. 1 Fig. 4a | Stromer 1931 p. 4 |
| *femur with spike like accessory trochanter* | UAT 678/20 | Stromer 1931, pl 1. Fig 14a  & Stromer 1936, p: 66 Abb. 9 D | Stromer 1931 p. 16 & Stromer 1934 p. 12, 35, 37 |
| *proximal fibula more anteriorly than posteriorly expanded* | UAT 678/20 | Stromer 1931, pl 1. Fig 15b  & Stromer 1936, p: 66 Abb. 9 D | Stromer 1931 p.18 |
| internasal suture inclined ventromedially* | /  No picture present in the respective view | Stromer 1931, pl 1. Fig 7b | Stromer 1931 p. 6 |
| anterior middle caudal vertebrae with well-developed pneumaticity including foramina posterior to prezygapophyses opening into spinoprezygpaophsyseal fossa* and pneumatic foramina in vertebral bodies | UAT 678/20 not clear enough in this part of the picture | Stromer 1931, pl 1. Fig 10a  Stromer 1936, p: 66 Abb. 9 D  Stromer 1936, p: 84 Abb. 20 A | Stromer 1931 p. 12;  Stromer 1934 p. 49, 51, 59 |

A) Should not be problematic at all, as observations on the photographs of the specimen are reproducible
B) This is the only point that could be considered slightly more problematic, as the characters given here are not clearly visible in any photograph of the specimen. However, these characters represent quite unusal morphologies and are thus hard to misinterpret (like the foramina in the caudal vertebrae). They are further mentioned multiple times throughout different publications, further strengthening our use of these characters. Nonetheless we did consider the critique and split these off into a separate section of the diagnosis.

Even if we were to take out the characters falling under section B, the specimen would still be diagnostic simply based on all characters directly observable in A. The following point *“If two distinct authors disagree on some of the statements by Stromer used here to define the new species, how could such disagreement be solved?”* is thus moot, as the main arguments used by us to define said species are based on verifiable evidence.

As for the second point:

*“Just because the Egyptian material cannot be referred to the same taxon of the Moroccan material (a conclusion I can endorse) does not automatically mean that the former could be used to erect a new taxon.”*

While this statement is generally true, a strong case can be made for the erection of a new taxon for the Egyptian carcharodontosaur. For one, the Egyptian taxon remains the only African carcharodontosaur with clearly associated cranial and postcranial elements, giving us the only source of information on the postcranial anatomy of the African members of this group. Furthermore, it remains one of the most informative African carcharodontosaurs, even with the specimen being destroyed. In our dataset, the Egyptian taxon is the second most completely coded African carcharodontosaur (78% missing data), after the neotype skull of *Carcharodontosaurus saharicus*, which is only slightly better coded (~76% missing data). All other African taxa in this group, and many of the named carcharodontosaurs for that matter, are significantly less complete (*C. iguidensis* 96% missing data, *Eocarcharia* ~94.5% missing data, *Lajasvenator* 86% missing data, *Lusovenator* 80% missing data, *Siamraptor* 82% missing data, *Taurovenator* 97.8% missing data, *Veterupristisaurus* 99% missing data). Even some of the better known South American taxa like *Tyrannotitan* only have slightly more phylogenetic information (77% missing data). In fact, the Egyptian carcharodontosaur is more completely coded than 33% of the OTUs used in our dataset. Given all of this we think it preferable to erect a new taxon, as we have already elaborated on further in our last Response to the reviewers.

Lastly, we want to address the statement of possible falsifiability of our taxonomic hypothesis:

*“Only a second specimen could falsify those words. But if the species is not a real taxon, such second specimen does not exist, and would never be found. In particular, in the case of non-existence of such taxon, no additional specimen actually exists, and we would never be able to say anything about the validity/revision of that taxon. In that case, the species would merely be a non-falsifiable hypothesis included in our taxonomies and phylogenies. This must be avoided.”*

This is an argument that, given the nature of morphospecies, is applicable not only to specimens that no longer exist but also to specimens that do still exist. Given the highly fragmentary nature of the fossil record, one can always make the argument that a given specimen is just an aberrant morphology of a known taxon. Unless a significantly large sample of fossils is found showing multiple transitionary forms between two different morphs (let’s say A and B), one can always make the argument, that A and B simply represent differing morphologies of the same taxon. Similarly, even if multiple specimens with the exact same morphologies of A and B are found but no transitionary forms, we still would not be able to confidently discredit an argument that we are simply still missing the transitionary forms. This is true whether or not we are referring to specimens that still exist or specimens that were destroyed. It is thus not a good argument against naming a species on no longer existing material.

“The authors listed a series of fossil taxa based on lost material in support of their preferred option (e.g., Maraapunisaurus). Yet, this is not a solid argument. First, I note that all of them are based on very fragmentary material (so, according to the conclusion of the manuscript, all of them would be considered nomina dubia even if based on accessible material). Second, all of the listed taxa are very problematic and barely mentioned in literature, thus of limited impact and significance. The exclusion of such taxa from the realm of the valid taxa would not impact in a significant way the progress of the palaeontological knowledge.”

We listed these taxa to underline our point that it is, in fact, possible to erect a new taxon based on no longer existing specimens. These examples were not meant as positive examples of such a practice, and we do agree with Reviewer 2 that these taxa should be considered nomina dubia even if they were based on accessible material. However, in our Manuscript we present 13 pages of detailed description of our proposed taxon, based both on a previous description by an author whose descriptions are generally considered to be accurate and on the illustrations (which, based on existing material figured by the same author, are probably also accurate) and on existing photographs of the specimen, which represents a substantial amount of information. This information is considerably more abundant than for the taxa mentioned above and, indeed, for many theropod dinosaurs known from still existing material, as outlined above. Disregarding this taxon from future phylogenies would be excluding a significant amount of information. (See also our previous comments addressing the second point)

“In conclusion, the main elements of the manuscript in its current form, excluding the erection of a new species, are sufficient for a solid and significant paper: removing any mention to a new taxon would not impact the relevance and importance of the manuscript, and would likely be considered more positively than a manuscript introducing such a controversial and problematic species actually based on a destroyed fossil and just a few photos.”

In theory, we agree with the main argument of this statement, that removing the erection of a new taxon and continuously referring to it with its specimen number would not significantly impact the relevance of this research in an ideal case. In practice, however, not naming this taxon would likely result in the following:

1. The Egyptian carcharodontosaur and its unique character combination would be more prone to be excluded from future phylogenies and other studies on the evolution, diversity and biogeography of carcharodontosaurid theropods. Unnamed species do not receive as much attention as named ones and are less likely to be considered in future works. If this happens, we lose a lot of anatomical information, and known carcharodontosaur diversity will be underestimated
2. As the reviewer himself admits, a convincing case is made that this specimen does represent a different taxon and (as shown above) it can be clearly diagnosed by a unique character combination and probably even several autapomorphic characters. Thus, it would be simply a matter of time before this taxon would be named by somebody.

**Reviewer 2:**

We thank reviewer 2 for his comments and corrected the respective typographical errors.

**In conclusion:**
We are acutely aware of the difficult situation we find ourselves in, and we acknowledge that erecting a new taxon for the Egyptian carcharodontosaur can be seen as problematic as the original specimen is no longer available. Nonetheless, we still think that making this material the type of a new taxon is the most preferable out of all the possible solutions for the tricky situation of this specimen, given the following points

- The Egyptian taxon represents one of the most complete African carcharodontosaurs known
- It is the only specimen out of eastern Africa and the only African specimen combining cranial and postcranial material
- The diagnosis of our species is based overwhelmingly on photographically documented and thus verifiable characters, even if we were to completely disregard Stromers descriptions and drawings
- If, as both reviewers agree, we made a convincing argument that the Egyptian carcharodontosaur represents a distinct taxon and can be diagnosed (as we now hope to have sufficiently demonstrated), it is only a matter of time before this taxon will be named.

On a related note, regarding the validity of using observations based on illustrations, photographs and descriptions for the diagnosis of a taxon:

Any referral to a now lost specimen, be it for the erection of a neotype, the referral of a taxon, or for simple comparative reasons has to be done without a specimen. This is common practice in paleontology and has been done for some of Stromers dinosaurs, like *Spinosaurus*, but also for comparisons of “*Carcharodontosaurus*”, the specific specimen in question here. If one questions the validity of illustrations, photographs and descriptions of a specimen, then as a consequence, we would have to disregard lost specimens completely. Any comparisons to still existing specimens, or even the referring of neotypes for lost specimens would be impossible, as there would be no way to falsify these taxonomic hypothesis with the original specimen being destroyed.

Stromers Spinosaurus, for example, has been commonly been referred to in the literature, used for comparsions to other spinosaurids, in phyologenies and has had a neotype proposed for it in 2014. If we discard all of Stromers writings as unfalsifiable evidence, we would consequently loose a good part of the information on this taxon and there would be no way to ever erect a neotype for this taxon either.

Consequentially, if one can use photographs, illustrations and descriptions to compare a taxon to a lost specimen, we do not see any convincing reason why one can not also use photographs, illustrations and descriptions to diagnose a new taxon, as it fundamentally requires the same comparative actions.

We hope that we now make a strong enough case for the erecting of a new taxon.
